# Metabolome and transcriptome analyses reveal molecular responses of two potato (*Solanum tuberosum* L.) cultivars to cold stress

**DOI:** 10.3389/fpls.2025.1543380

**Published:** 2025-04-25

**Authors:** Xiang Li, Zhenzhen Zheng, Yun Zhou, Shenglong Yang, Wang Su, Heng Guo, Guangji Ye, Jian Wang

**Affiliations:** ^1^ Academy of Agriculture and Forestry Sciences, Qinghai University, Xining, China; ^2^ Qinghai Provincial Key Laboratory of Potato Breeding, Qinghai University, Xining, China; ^3^ Ministry of Education Engineering Research Center of Potato in Northwest Region, Qinghai University, Xining, China

**Keywords:** potato (*Solanum tuberosum L.*), cold stress (CS), differentially accumulated metabolites(DAMs), differentially expressed genes (DEGs), KEGG pathway

## Abstract

**Introduction:**

Potato (*Solanum tuberosum* L.), as an important food crop on the Qinghai-Tibet Plateau, is prone to low temperature and frost damage during the seedling stage, causing economic losses for farmers.

**Methods:**

In this study, metabolome and transcriptome analyses were conducted on the leaves of Atlantic (cold-resistant) and KY140 (cold-sensitive) potato varieties following exposure to cold stress (CS).

**Results:**

After CS, 298 and 195 differentially accumulated metabolites (DAMs) were identified in Atlantic and KY140, respectively, with 124 common DAMs, including lipids, flavonoids, alkaloids, organic acids, amino acids and their derivatives, nucleotides and their derivatives, lignans and coumarins, phenolic acids, and terpenoids. A total of 6928 and 2428 differentially expressed genes(DEGs) were identified in Atlantic and KY140, respectively, with 1131 common DEGs. Joint analysis of DAMs and DEGs, “flavonoid-related metabolism,” “lipid metabolism,” and “amino acid metabolism” were plotted. Cinnamic acid, caffeic acid, naringenin, and γ-aminobutyric acid (GABA) might participate in the regulation of potato resistance to CS. The genes *StPAL(Soltu.Atl.09_2G005110)* and *StGAD(Soltu.Atl.11_3G000340)* encode enzymes responsible for the biosynthesis of cinnamic acid and GABA, respectively, suggesting their involvement in the regulation of cold resistance in potato.

**Discussion:**

Our results provided novel insights into the molecular mechanisms underlying cold resistance in potato.

## 1 Introduction

Potato (*Solanum tuberosum* L.) is an annual herbaceous plant belonging to the family *Solanaceae* and the genus *Solanum*. Potatoes are rich in starch, protein, minerals, vitamins, and dietary fiber, imparting the advantages of high yield, rich nutrition, good strength, and easy digestibility. It is a versatile crop that can be used as food, vegetables, feed, and industrial raw materials (Yang et al., 2014; Liu J. T. et al., 2020). Low temperatures and frost not only affect the growth and development of potato but can also cause potato death in severe cases, leading to a significant reduction in yield and causing serious economic losses for potato growers (Liu J. T. et al., 2020).

To minimize the cold stress (CS)-induced damage, potato has evolved a series of adaptive mechanisms. Perception of low temperature by plants is extremely complex. The AP2/ERF transcription factors (TFs) are located at the center of the entire molecular regulatory network underlying plant responses to CS (Feng et al., 2020). Potato usually resists CS by producing various functional molecules or changing the state of some molecules. Increasing the unsaturation of fatty acids can improve the fluidity of plant cell membranes, thereby increasing the plant’s tolerance to low temperatures (Uemura and Steponkus, 1997; Gao et al., 2015; Hou et al., 2016). The antioxidant enzyme system in plant cells plays a key role in resisting CS. Enzymes such as peroxidase (POD), catalase (CAT), and superoxide dismutase (SOD) effectively eliminate free radicals in cells and reduce the probability of cell damage (Sun et al., 2006). High POD, CAT, and SOD activities can enhance the cold resistance of potato (Xu et al., 2016; Wang M. X. et al., 2021; Duan et al., 2022). Additionally, potato can reduce water potential and water loss and maintain osmotic balance inside the cells through their own osmotic regulatory compounds, which helps them maintain normal morphology and physiological functions and enhance their cold resistance (Liu J. T. et al., 2020). High proline and soluble protein and sugar contents can also enhance the cold resistance of potato (Xu et al., 2016; Wang M. X. et al., 2021; Duan et al., 2022).

Metabolomics, a link between genomics and phenotype, is an important omics technology employed for studying systems biology (Duan, 2019). Cook et al. (2004) found that the *Arabidopsis* metabolome is extensively reconfigured in response to low temperatures, with the C-repeat binding factor (CBF) cold response pathway playing an important role in this process. Metabolome analysis of winter rapeseed cultivar ‘Longyou 7’ (*Brassica campestris* L.) roots under CS revealed that most of the differentially accumulated metabolites (DAMs) were enriched in several Kyoto Encyclopedia of Genes and Genomes (KEGG) pathways such as “fructose and mannose metabolism,” “starch and sucrose metabolism,” “arachidonic acid metabolism,” “phenylalanine metabolism,” “glycerophospholipid metabolism,” “galactose metabolism,” “α-linolenic acid metabolism,” “amino acid biosynthesis,” “arginine and proline metabolism,” and “cysteine and methionine metabolism” (Fang et al., 2021). Transcriptomic technologies have been widely used to study changes in gene expression in plants under low-temperature conditions. Transcriptomic analysis of maize roots subjected to CS revealed a total of 189 lipid-related differentially expressed genes (DEGs), which were annotated and classified into various lipid metabolism pathways. Most of these DEGs were enriched in “eukaryotic phospholipid synthesis,” “fatty acid elongation,” and “phospholipid signaling” (Zhao et al., 2021). Transcriptome analysis of low temperature-treated rice (*Oryza sativa* L.) identified 55 TF families, including AP2/ERF-ERF, bHLH, bZIP, MYB, and NAC, and revealed that most DEGs were enriched in “lipid metabolism,” “carbohydrate metabolism,” “amino acid metabolism,” “MAPK signaling pathway,” and “plant-pathogen interaction” (Liu et al., 2022). The regulation of phenotype by genes can be intuitively reflected in the changes in the types and levels of metabolites in plants. The combined metabolome and transcriptome analysis can cross-validate the two sets of data, increasing the reliability of the results. At present, such combined analysis is widely used to study the resistance mechanism of plants under low-temperature stress. The comprehensive metabolome and transcriptome analysis of Jimai 325 and its mutant MU-134 under CS showed that the cold resistance of wheat is closely related to seven upregulated energy metabolites and eight upregulated enzymes involved in the sucrose and amino acid biosynthesis pathway. In addition, 13 metabolites and 14 key enzymes that were involved in the flavonol biosynthesis pathway were identified (Lv et al., 2022). Li et al. (2023) performed a combined metabolome and transcriptome analysis of the cold-tolerant ‘Donghe No.1’ and cold-sensitive ‘21^st^ Century’ peach cultivars under low-temperature treatment and identified “galactose metabolism,” “phenylpropanoid biosynthesis,” and “flavonoid biosynthesis” as the key pathways involved in peach cold resistance. Wang X. et al. (2021) conducted a comprehensive analysis of the metabolome and transcriptome data of SLH (cold-resistant) and ZH12 (cold-sensitive) peanut varieties under low-temperature treatment, revealing commonly enriched pathways such as “carbohydrate metabolism.”

In recent years, studies on the metabolome and transcriptome mechanisms underlying CS in potato are limited (Kou et al., 2018; Zhang, 2023; Zheng et al., 2024). So, the integrated omics analysis (metabolome and transcriptome) of potato under CS could prove to be a fascinating approach to revealing key CS-responsive mechanisms at the metabolic and molecular levels. The cold resistance of 15 potato materials was evaluated, with Atlantic showing the strongest cold resistance and KY140 showing the weakest cold resistance. We performed metabolomic and transcriptomic analyses of the leaves obtained from Atlantic (cold-resistant) and KY140 (cold-sensitive), under control (25°C) and CS (−4°C, 12 h) conditions, aiming to reveal the metabolites, key genes, and pathways in the response to CS between the two cultivars. The experimental materials used in this study were tissue culture seedlings, which are cultured under laboratory conditions, occupying less area and not being affected by seasons and climate, thus allowing continuous production throughout the year. The experimental conditions (−4°C treatment) were, strictly speaking, frost damage. This study suggests that “flavonoid-related metabolism,” “lipid metabolism,” and “amino acid metabolism” play important functions in the process of potato responding to CS. Our results provided novel insights into the mechanisms underlying CS adaptation in potato, thus facilitating the development of CS-tolerant potato varieties.

## 2 Materials and methods

### 2.1 Plant growth conditions and stress treatments

For this study, Atlantic and KY140 tissue culture seedlings (virus-free) were cultured on MS medium for 18 days. The light was about 2500 lx, and the light duration was 16 h per day. The control and treatment groups were subjected to temperatures of 25°C (CK) and −4°C, respectively, for 7 days. Then, all the samples were grown at 25°C for one day for phenotypic difference identification. After leaf damage, it affects the normal growth of plants. A leaf was considered damaged if it was wilted by half or more. The damaged rate of the leaves in a stressed plant was the number of damaged leaves divided by the total number of leaves in the stressed plant. Both Atlantic and KY140 each plant has different degrees of damage. The damage rate of the leaves in Atlantic and KY140 are the averages of the damage rate of 60 cold-stressed plants, respectively. The control group (CK, 25°C) and the treated groups (6, 12, and 18 h; −4°C) were used for the determination of physiological indicators. CK and the treated group (12 h, −4°C) were used for metabolome and transcriptome analysis. All treatments were performed with three biological replicates.

### 2.2 Determination of physiological parameters

POD, CAT, and SOD activities and proline and soluble protein and sugar contents were analyzed using respective kits (Nanjing Jiancheng Biology Co., Ltd., Nanjing, China).

### 2.3 Omics analysis

Library construction and RNA sequencing were performed by MetWare (Wuhan, China). The metware database (MWDB) (Metware Biotechnology Co., Ltd., Wuhan, China) was used for metabolite identification. The criteria used to screen for DAMs were as follows: Variable importance in projection (VIP) ≥ 1 and fold change (FC) ≥ 2 or FC ≤ 0.5. The criteria used to screen for DEGs were as follows: |log_2_FC| ≥ 1 and false discovery rate (FDR) < 0.05. Screening of DAMs that might participate in the regulation of potato resistance: (1) When the level of Atlantic_vs_CS-Atlantic DAMs content increased, and the level of KY140_vs_CS-KY140 DAMs content increased or decreased, the Atlantic_vs_CS-Atlantic_Log_2_FC was higher than the KY140_vs_CS-KY140_Log_2_FC. (2) When the level of Atlantic_vs_CS-Atlantic and KY140_vs_CS-KY140 DAMs content both decreased, Atlantic_vs_CS-Atlantic_|Log_2_FC| was higher than the KY140_vs_CS-KY140_|Log_2_FC|. Screening of DEGs that might involve in the regulation of cold resistance in potato: (1) When the level of Atlantic_vs_CS-Atlantic DEGs expression increased, and the level of KY140_vs_CS-KY140 DEGs expression increased or decreased, the Atlantic_vs_CS-Atlantic_Log_2_FC was higher than the KY140_vs_CS-KY140_Log_2_FC. (2) When the level of Atlantic_vs_CS-Atlantic and KY140_vs_CS-KY140 DEGs expression both decreased, Atlantic_vs_CS-Atlantic_|Log_2_FC| was higher than the KY140_vs_CS-KY140_|Log_2_FC|.

### 2.4 Quantitative real-time polymerase chain reaction (qRT-PCR) analysis

Total RNAs were extracted from the leaf samples with RNAprep Pure polysaccharide and polyphenol plant total RNA extraction kit (Tiangen Biotech Co., Ltd., Beijing, China) and then reverse transcribed into cDNA with PrimeScript™ RT Master Mix (Perfect Real Time) (TaKaRa Biotechnology Co., Ltd., Dalian, China). qRT-PCR was carried out using TB Green^®^ Premix Ex Taq™ II (Tli RNaseH Plus) (TaKaRa Biotechnology Co., Ltd., Dalian, China) on Roche LightCycler® 96 qRT-PCR System (Basel, Switzerland). The results of qRT-PCR were obtained using the 2^−ΔΔCt^ method.

### 2.5 Data analysis

Data Summary with Office Excel 2019 was used for the analysis of phenotypic data, physiological index data, and qRT-PCR data. GraphPad Prism 8 was used for bar charts and significance analysis. Statistical significance was determined by one-way ANOVA and Tukey’s multiple comparisons test.

## 3 Results

### 3.1 Phenotypic variation in potato under CS

**Figure 1:**
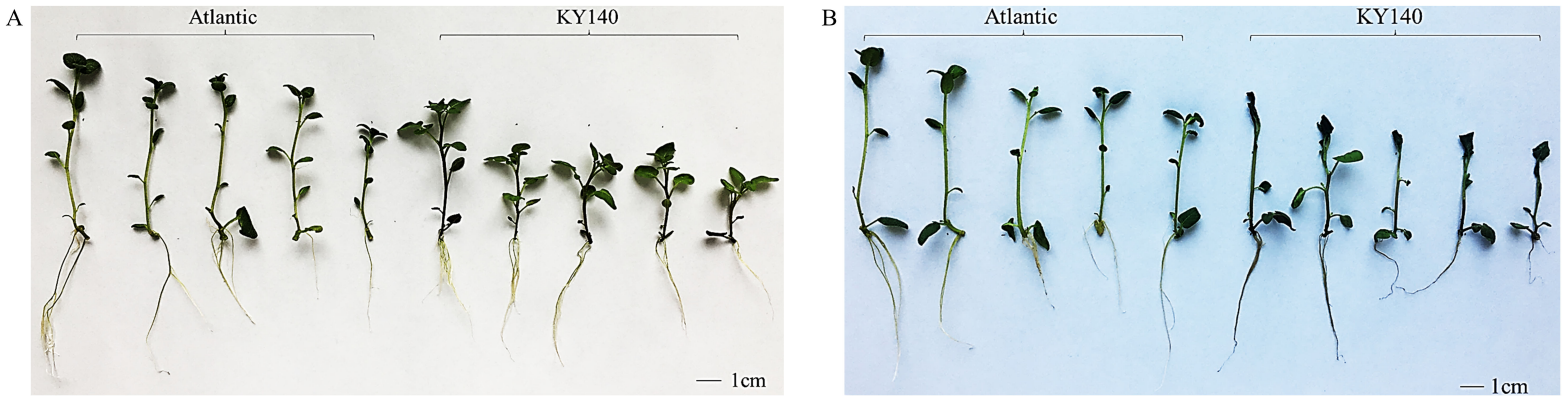
Phenotypic characteristics of Atlantic and KY140 under cold stress (CS, −4°C). **(A)** Shoot morphology (control group, CK). **(B)** Shoot morphology (treated group).

After being subjected to CS for 7 days and then allowed to recover at 25°C for one day, 12.2% and 57.1% of the Atlantic and KY140 leaves damaged, respectively (**Figures 1A, B**).

### 3.2 Changes in the physiological indicators of potato under CS

**Figure 2:**
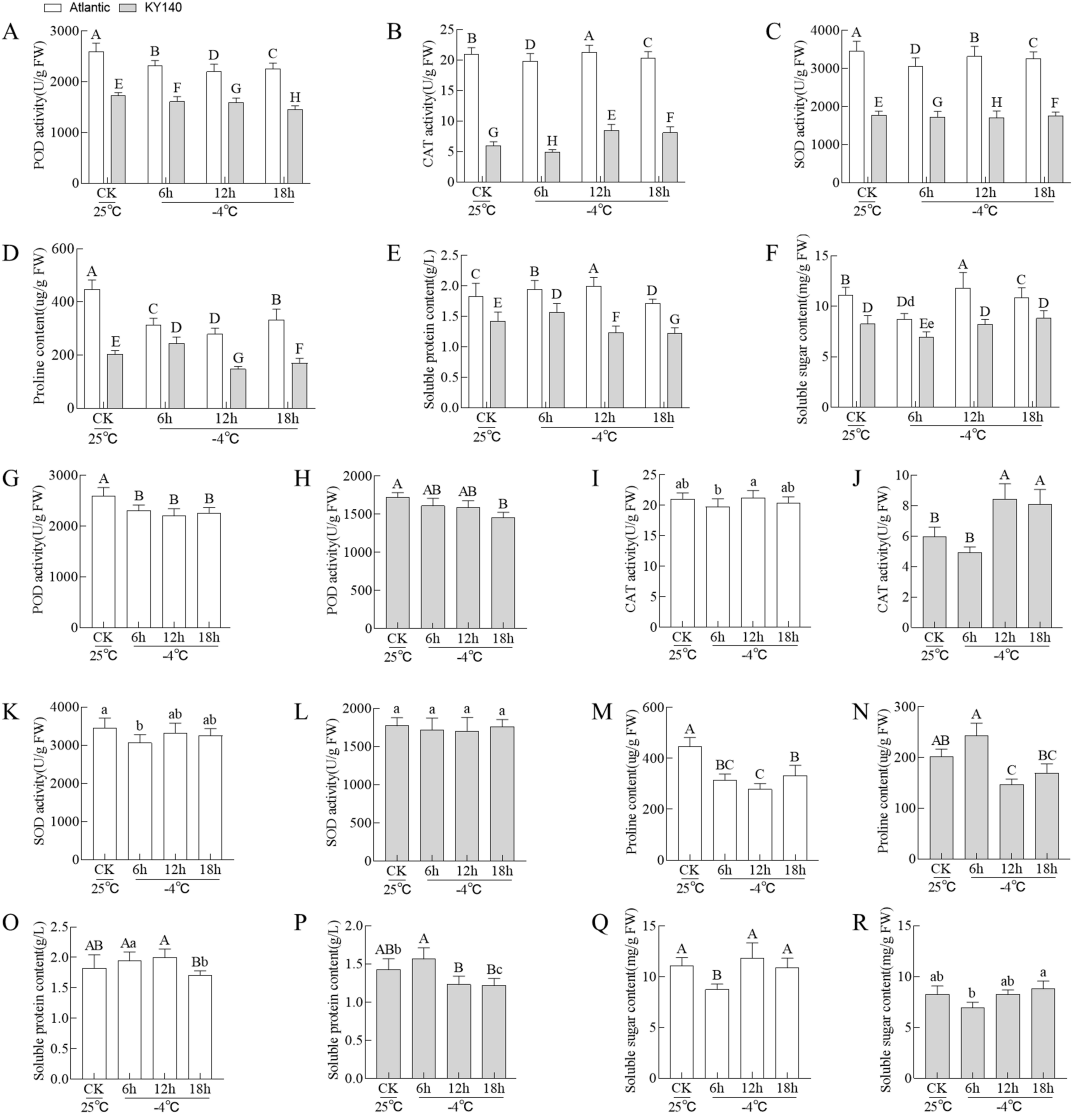
Physiological characteristics of Atlantic and KY140 under cold stress (CS, −4°C). **(A–F)** Comparison of between-group differences. **(G–R)** Comparison of within-group differences. All treatments were performed with at least six biological replicates ± standard deviation (SD). Bar charts with different capital/small letters indicating significant differences (P < 0.01/P < 0.05). When both capital and small letters are present, the small letters should be used as the main comparison. For instance, in panel **(F)**, when comparing the −4°C treatment of Atlantic and KY140 for 6 h, the comparison should be made between “d” and “e.” The correlation was significant at P < 0.05.

Under different treatments, Atlantic exhibited higher POD, CAT, and SOD activities and proline and soluble protein and sugar contents than KY140 (**Figures 2A–R**). Furthermore, among the two Atlantic groups, CAT activity and soluble protein and sugar contents peaked at 12 h and were higher in the treated group than the corresponding CK. Among the two KY140 groups, SOD activity and proline content reached their lowest values at 12 h, and these values were lower in the treated group than the corresponding CK. The unique cold tolerances of these two species made them ideal models for studying the potential mechanisms underlying cold tolerance in potato.

### 3.3 Global metabolomic changes in potato under CS

**Figure 3:**
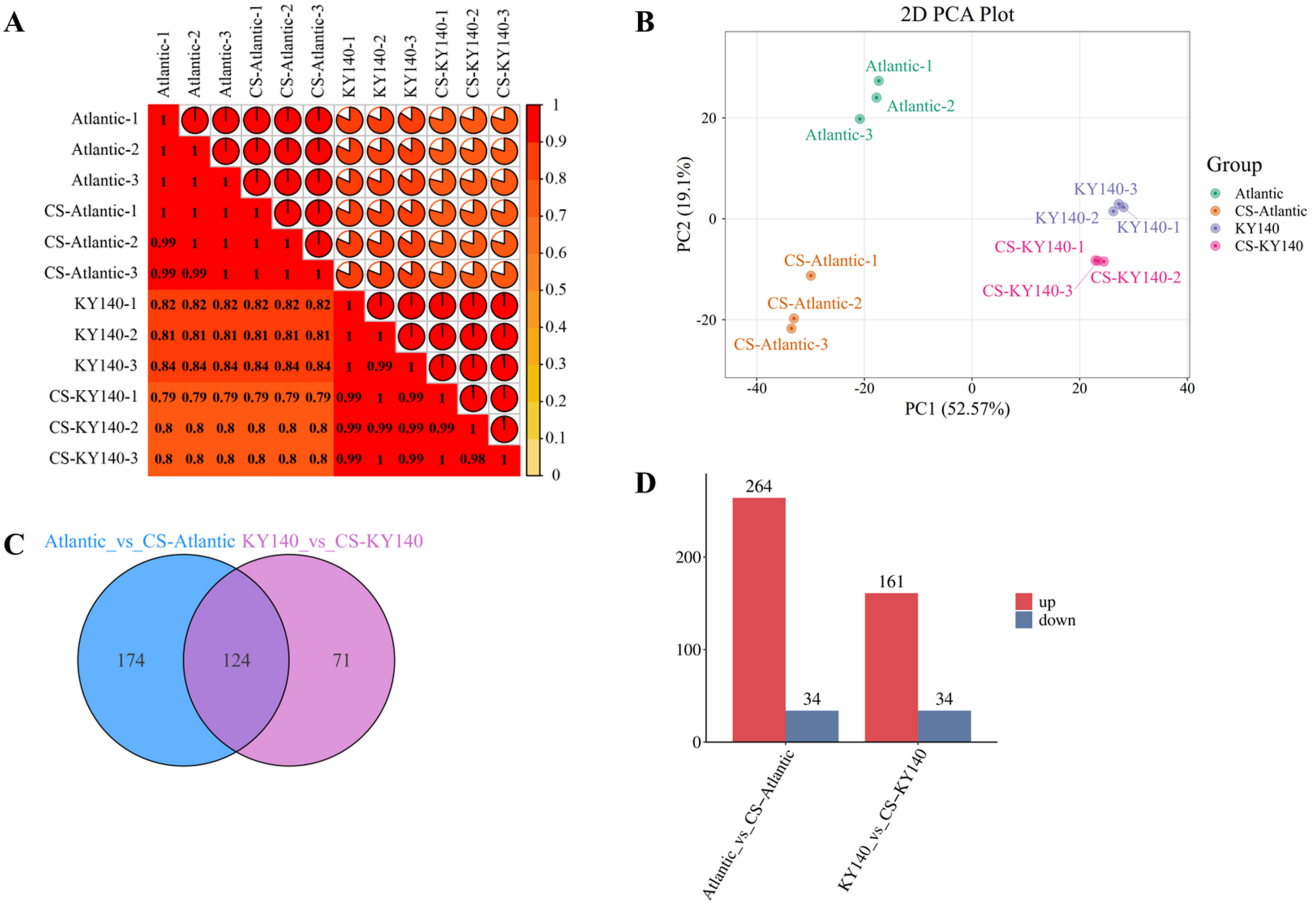
Metabolome of potato in response to cold stress (CS). **(A)** Correlation analysis of all samples. **(B)** Principal component analysis (PCA) of metabolomics results. **(C)** Venn diagram of differentially accumulated metabolites (DAMs) among Atlantic_VS_CS-Atlantic and KY140_VS_CS-KY140. **(D)** Number of DAMs between Atlantic_VS_CS-Atlantic and KY140_VS_CS-KY140. Atlantic, untreated Atlantic; CS-Atlantic, treated Atlantic; KY140, untreated KY140; CS-KY140, treated KY140.

The correlation analysis reveals that the intra-group sample correlations are significantly high, which underscores the robust reproducibility and reliability of the metabolomic data (**Figure 3A**). Principal component analysis (PCA) revealed clear separation among different material groups, with minimal dispersion within each group (**Figure 3B**). These results demonstrated that the data met the requirements for subsequent in-depth analysis (**Figures 3A, B**). We identified 298 and 195 DAMs in Atlantic and KY140, respectively, with 124 common DAMs (**Figures 3C, D**), including lipids, flavonoids, alkaloids, organic acids, amino acids and their derivatives, nucleotides and their derivatives, lignans and coumarins, phenolic acids, and terpenoids. Among the identified DAMs, 264 and 161 were upregulated and 34 and 34 were downregulated in Atlantic and KY140, respectively, indicating significantly more upregulated than downregulated DAMs in both genotypes (**Figure 3D**). Furthermore, Atlantic harbored more upregulated DAMs than KY140 (**Figure 3D**). Among the treated groups Atlantic harbored a higher number of unique DAMs (n = 174) was than KY140 (n = 71), suggesting that more DAMs were involved in regulating CS resistance in Atlantic than in KY140 (**Figure 3C**).

### 3.4 Global transcriptomic changes in potato under CS

**Figure 4:**
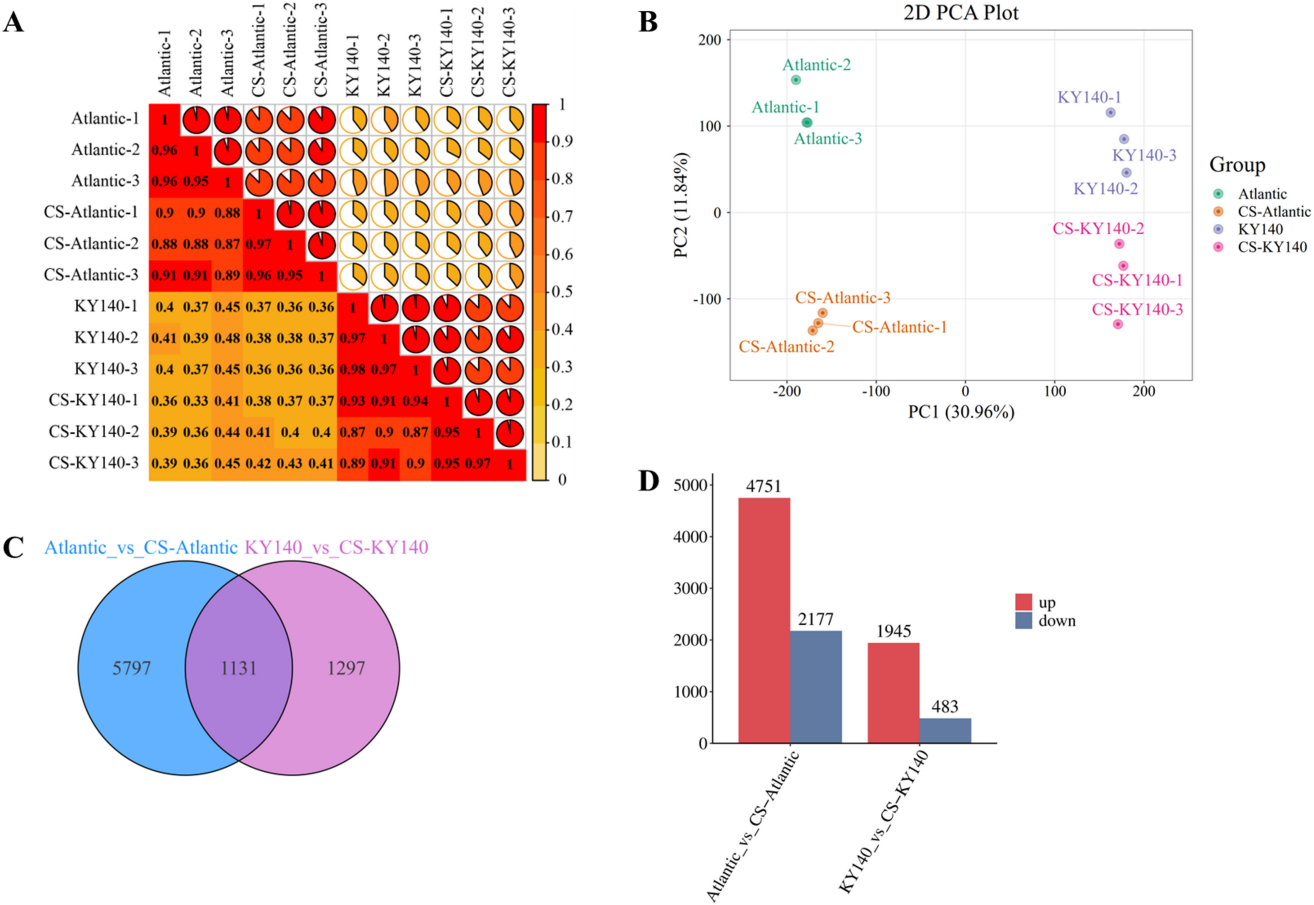
Transcriptome of potato in response to cold stress (CS). **(A)** Correlation analysis of all samples. **(B)** Principal component analysis (PCA) of transcriptomics results. **(C)** Venn diagram of differentially expressed genes (DEGs) between Atlantic_VS_CS-Atlantic and KY140_VS_CS-KY140. **(D)** Number of DEGs between Atlantic_VS_CS-Atlantic and KY140_VS_CS-KY140. Atlantic, untreated Atlantic; CS-Atlantic, treated Atlantic; KY140, untreated KY140; CS-KY140, treated KY140.

A total of 8225 DEGs were identified in Atlantic_VS_CS-Atlantic and KY140_VS_CS-KY140 under CS, including 1131 common DEGs (**Figures 4C, D**). The high intra-group sample correlations observed in the correlation analysis demonstrate the strong reproducibility and reliability of the sequencing data (**Figure 4A**). PCA revealed clear separation among different groups, with minimal dispersion within each group (**Figure 4B**). Furthermore, 6928 and 2428 DEGs were identified in Atlantic_VS_CS-Atlantic and KY140_VS_CS-KY140, respectively (**Figures 4C, D**). Among them, 4751 and 1945 DEGs were upregulated and 2177 and 483 DEGs were downregulated in Atlantic_VS_CS-Atlantic and KY140_VS_CS-KY140, respectively (**Figure 4D**). Thus, the number of upregulated DEGs in both groups was significantly higher than that of downregulated DEGs (**Figure 4D**). Among the treated groups, Atlantic harbored a higher number of unique DEGs (n = 5797) than KY140 (n = 1297), more DEGs were involved in cold regulation in Atlantic (**Figure 4C**). These results indicated that the expression patterns of differential genes in the two genotypes might impact the CS tolerance of the varieties.

### 3.5 Identification and expression analysis of TFs related to CS

**Figure 5:**
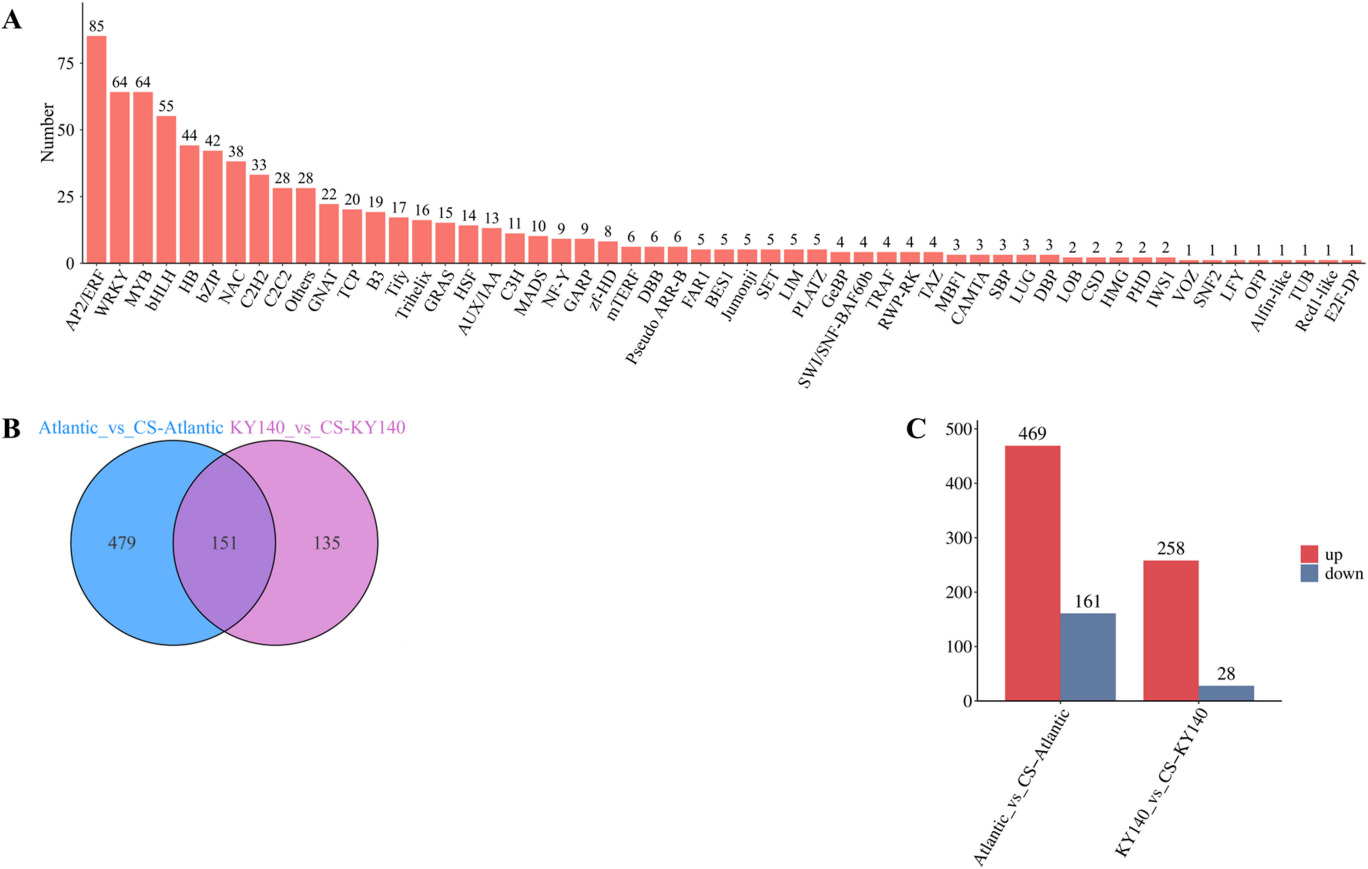
Analysis of transcription factors (TFs) of Atlantic_VS_CS-Atlantic and KY140_VS_CS-KY140. **(A)** Bar chart of TF classes. **(B)** Venn diagram of differentially expressed TFs between Atlantic_VS_CS-Atlantic and KY140_VS_CS-KY140. **(C)** TF statistics chart. Atlantic, untreated Atlantic; CS-Atlantic, treated Atlantic; KY140, untreated KY140; CS-KY140, treated KY140.

TFs play a crucial regulatory role in potato cold resistance. In this study, a total of 765 TFs were identified as hypothetical regulators of cold tolerance in potato, mainly belonging to the AP2/ERF, WRKY, MYB, bHLH, HB, bZIP, and NAC families (n = 85, 64, 64, 55, 44, 42, and 38, respectively), distributed across 55 gene families (**Figure 5A**). Apparently, AP2/ERF, WRKY, MYB, and bHLH family TFs were the key TFs associated with the cold resistance of both varieties. Among the treated groups, 469 and 258 upregulated and 161 and 28 downregulated TFs were detected in Atlantic and KY140, respectively (**Figure 5C**). Thus, both varieties harbored significantly more upregulated TFs than downregulated TFs (**Figure 5C**). Moreover, among the treated groups, Atlantic harbored more differentially expressed TFs (n = 479) than KY140 (n = 135), indicating that more TFs were involved in cold regulation in Atlantic than in KY140 (**Figure 5B**). Furthermore, the numbers of CS-activated TFs markedly varied between the two varieties, suggesting distinct gene regulatory networks.

### 3.6 qRT-PCR validation

**Figure 6:**
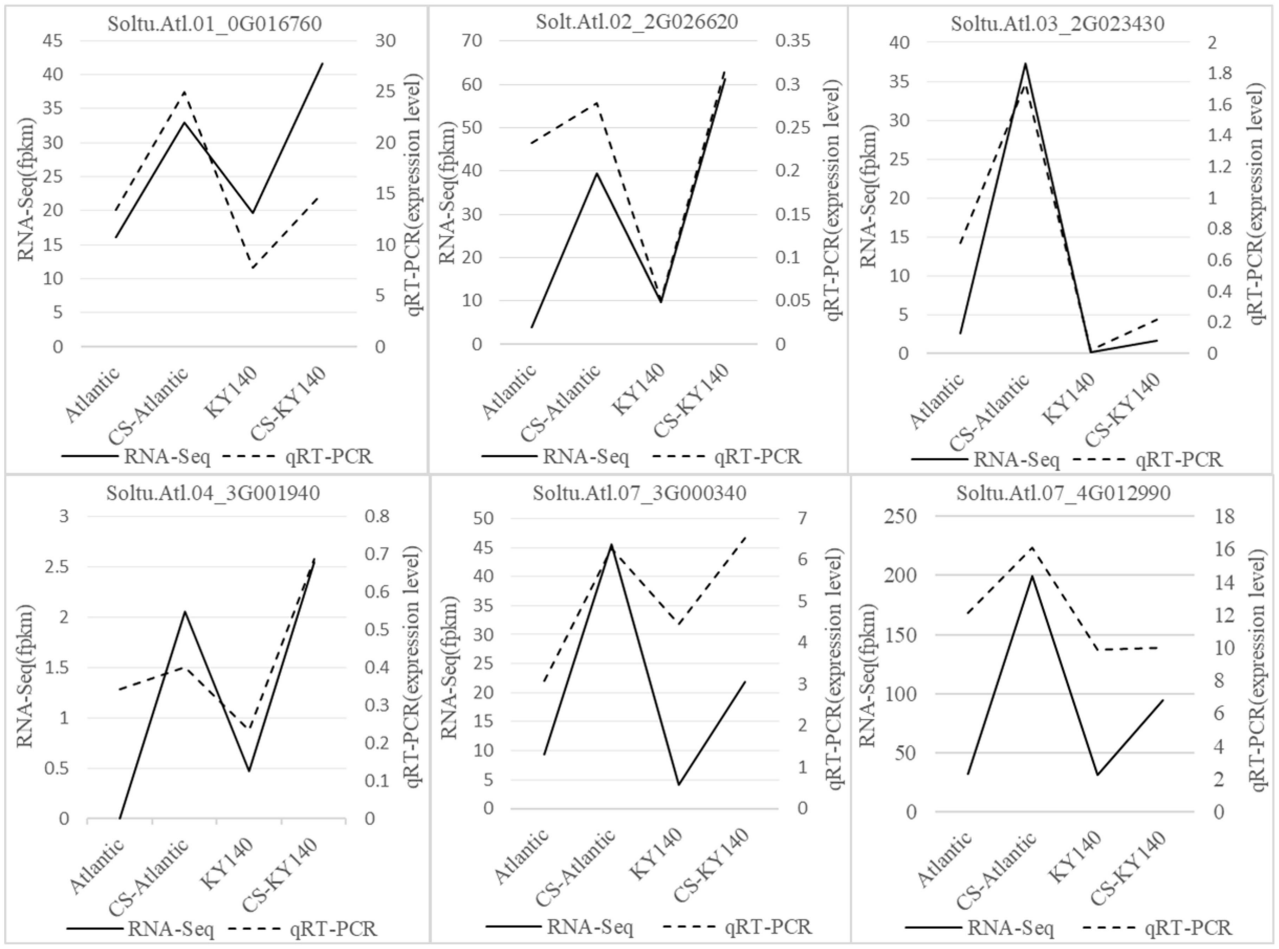
Expression patterns of six differentially expressed genes (DEGs) in potato under cold stress (CS).

To verify the reliability of the RNA-Seq data, six randomly selected cold tolerance-related DEGs were analyzed using qRT-PCR. The expression trends of the six DEGs observed through qRT-PCR were highly consistent with the RNA-Seq data (**Figure 6**), indicating the high reliability of the RNA-Seq data.

### 3.7 Gene ontology (GO) analysis of the DEGs related to CS

**Figure 7:**
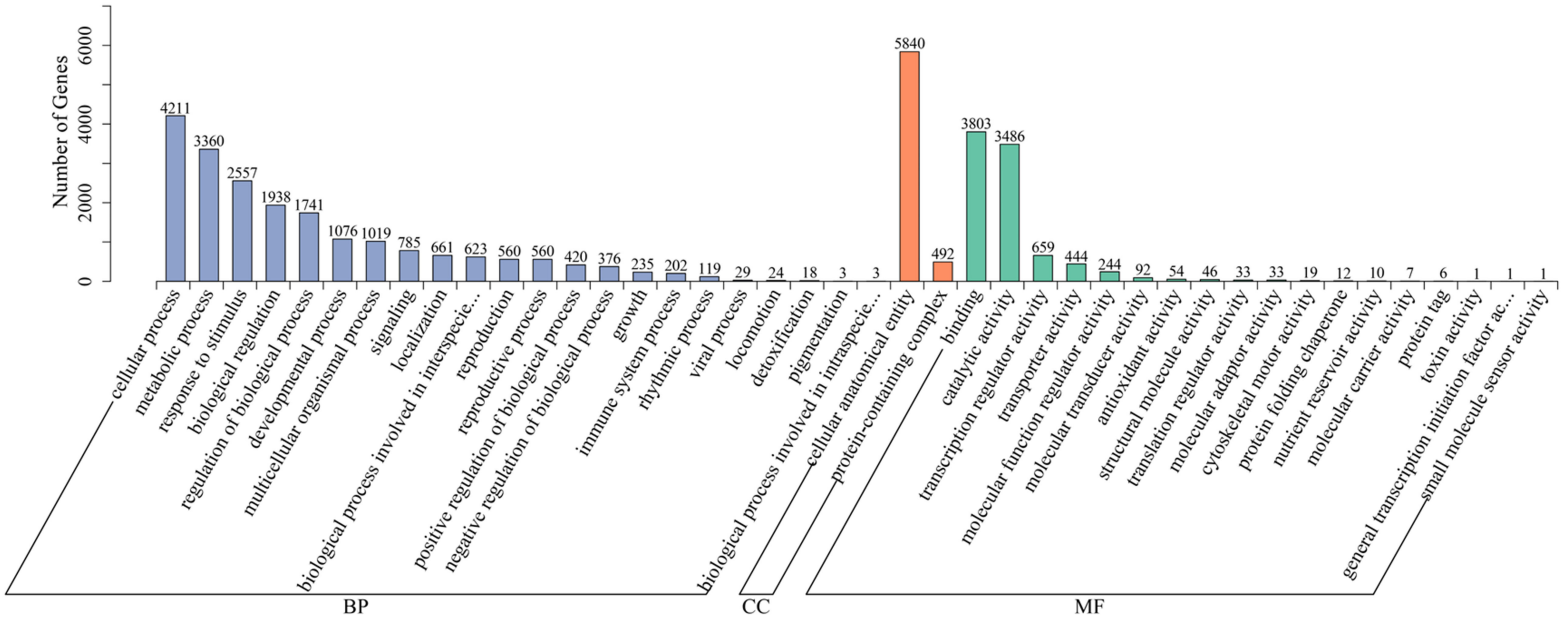
Gene ontology (GO) classification of differentially expressed genes (DEGs) in Atlantic_vs_CS-Atlantic and KY140_vs_CS-KY140. Atlantic, untreated Atlantic; CS-Atlantic, treated Atlantic; KY140, untreated KY140; CS-KY140, treated KY140.

Next, we explored the potential functions of all DEGs in Atlantic_vs_CS-Atlantic and KY140_vs_CS-KY140 using the GO database. Only 6869 out of the identified 8225 DEGs were annotated to GO terms, and the annotated DEGs were categorized into 42 GO terms, including “biological process,” “molecular function,” and “cellular component” (**Figure 7**). In “biological process,” most DEGs were annotated to “cellular process” and “metabolic process.” In “molecular function,” most DEGs were annotated to “binding” and “catalytic activity.” In “cellular component,” the DEGs were only annotated to “protein-containing complex” and “cellular anatomical entity.” These results indicated that these pathways played an important role in the response of potato to CS.

### 3.8 Combined metabolome and transcriptome analysis of KEGG pathways in potato subjected to CS

**Figure 8:**
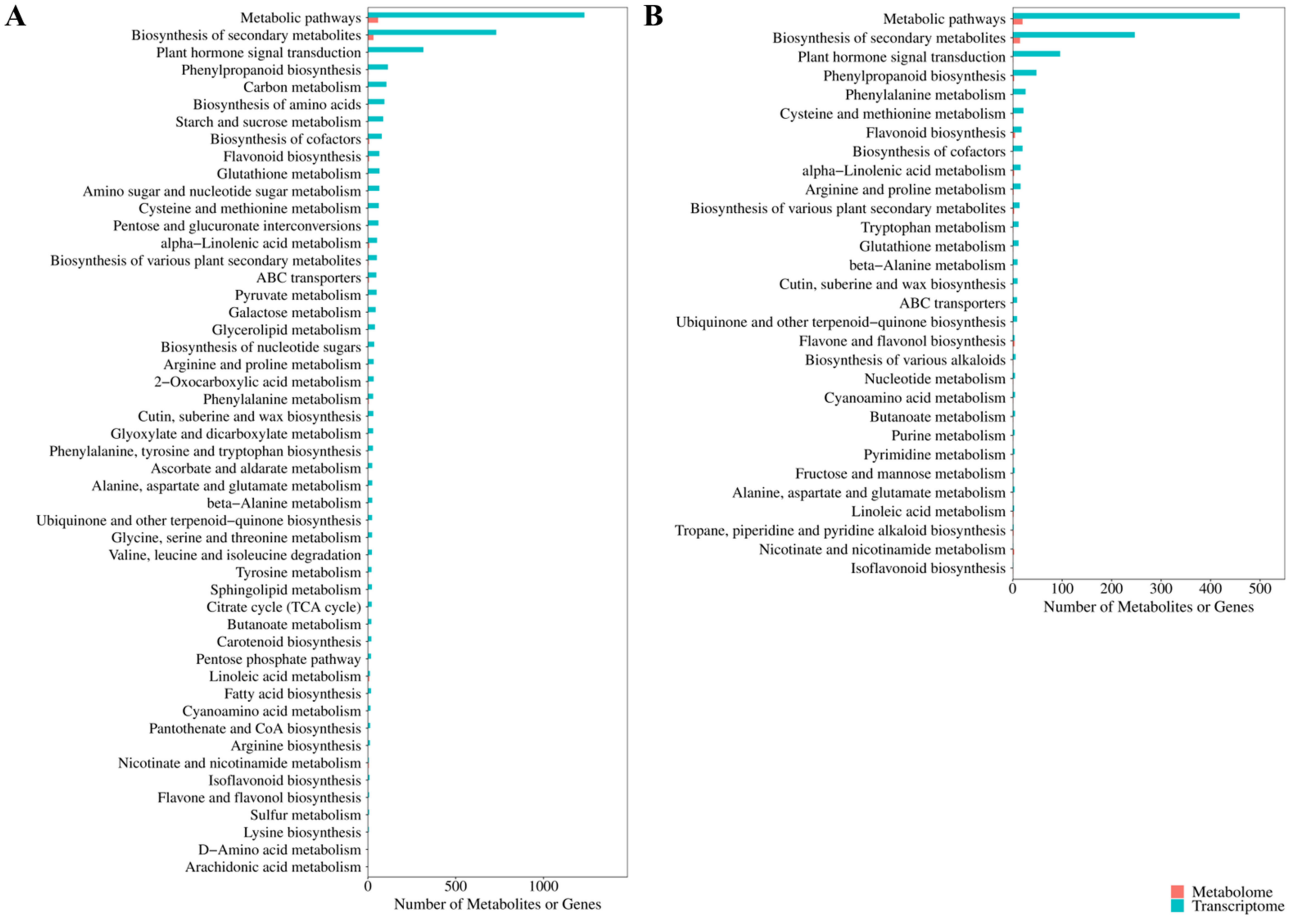
Kyoto Encyclopedia of Genes and Genomes (KEGG) pathway analysis of differentially accumulated metabolites (DAMs) and differentially expressed genes (DEGs). **(A)** Atlantic_vs_CS-Atlantic DAMs and DEGs common_KEGG_enrichment_bar. **(B)** KY140_vs_CS-KY140 DAMs and DEGs common_KEGG_enrichment_bar. A bar chart was created using the commonly enriched KEGG pathways from both omics groups. The bar chart shows the number of DAMs and DEGs enriched in each pathway. For cases where the number of common KEGG pathways exceeded 50, only the top 50 ranked by P-value are displayed, with the transcriptome as the reference. If there are fewer than 50 enriched pathways, all of them are displayed. The x-axis represents the number of DAMs or DEGs annotated to the pathway, and the y-axis represents the name of the KEGG pathway.

In order to further elucidate the regulatory mechanisms of potato cold resistance, the DAMs and DEGs were together subjected to KEGG pathway enrichment (**Figures 8A, B**). In Atlantic_VS_CS-Atlantic, the DAMs and DEGs were associated with 63 and 143 KEGG pathways, respectively, with a total of 63 common KEGG pathways. In KY140_vs_CS-KY140, the DAMs and DEGs were associated with 31 and 129 KEGG pathways, respectively, with a total of 30 common KEGG pathways. Among the treated groups, Atlantic exhibited activation of more KEGG pathways, with the induction of more DAMs and DEGs than KY140. These pathways primarily included “flavonoid-related metabolism,” “lipid metabolism,” “amino acid metabolism,” “carbohydrate metabolism,” “nucleotide metabolism,” and “energy metabolism.”

### 3.9 “Flavonoid-related metabolism” in potato under CS

**Figure 9:**
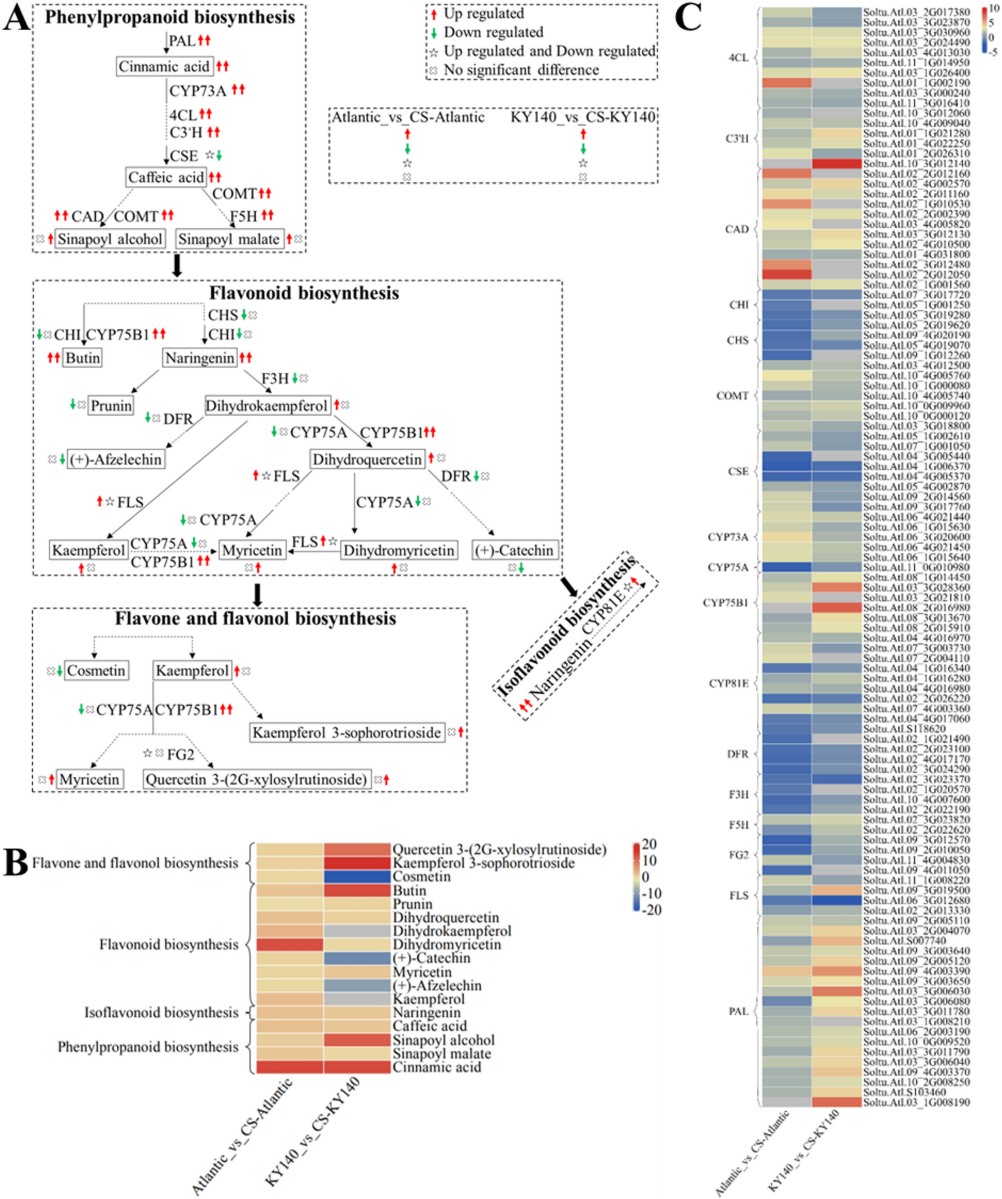
Metabolome and transcriptome profiling of differentially accumulated metabolites (DAMs) and differentially expressed genes (DEGs) associated with “flavonoid-related metabolism” in potato under cold stress (CS). **(A)** Comprehensive analysis of DAMs and related key enzymes in “flavonoid-related metabolism.” The ends of the arrow-headed lines represent DAMs, and the onlines represent enzymes. A solid line between two DAMs indicates a direct connection, while a dashed line indicates that the two DAMs are not directly connected but have other DAMs in between. PAL, Phenylalanine ammonia-lyase; C3’H, 5-O-(4-coumaroyl)-D-quinate 3’-monooxygenase; F5H, Ferulate-5-hydroxylase; CYP73A, Trans-cinnamate 4-monooxygenase; CSE, Caffeoyl shikimate esterase; COMT, Caffeic acid 3-O-methyltransferase; 4CL, 4-Coumarate: CoA ligase; CAD, Cinnamyl-alcohol dehydrogenase; F3H, Flavanone-3-hydroxylase; DFR, Bifunctional dihydroflavonol 4-reductase/flavanone 4-reductase; FLS, Flavonol synthase; CYP75B1, Flavonoid 3’-monooxygenase; CHI, Chalcone isomerase; CHS, Chalcone synthase; CYP75A, Flavonoid 3’,5’-hydroxylase; CYP81E, Isoflavone/4’-methoxyisoflavone 2’-hydroxylase; and FG2, Flavonol-3-O-glucoside L-rhamnosyltransferase. DAMs and DEGs were investigated using the Kyoto Encyclopedia of Genes and Genomes (KEGG) to map the possible KEGG pathway maps for the biological interpretation of systemic functions (https://www.kegg.jp/kegg/pathway.html). **(B)** Heatmap of DAMs in “flavonoid-related metabolism.” The x-axis represents Atlantic_vs_CS-Atlantic_Log_2_FC and KY140_vs_CS-KY140_Log_2_FC. When DAMs appear in multiple pathways, only one of the pathways is displayed, and the colored bar in the upper right corner represents the contents of DAMs, with red color indicating high-content DAMs and blue color indicating low-content DAMs. **(C)** Heatmap of DEGs in “flavonoid-related metabolism.” The x-axis represents Atlantic_vs_CS-Atlantic_Log_2_FC and KY140_vs_CS-KY140_Log_2_FC. A color bar is shown in the upper right corner, with colors ranging from blue to red indicating the expression levels of DEGs from low to high.

To better analyze the synergy between DAMs and DEGs, we selected several DAMs and KEGG pathways related to “flavonoid-related metabolism” and constructed a schematic map depicting the differences between Atlantic and KY140 after CS (**Figure 9**). Both Atlantic_vs_CS-Atlantic and KY140_vs_CS-KY140 exhibited elevated levels of cinnamic acid, caffeic acid, and naringenin, suggesting that they might participate in the regulation of potato resistance to CS. Cinnamic acid, butin, sinapoyl alcohol, kaempferol 3-O-sophorotrioside, and quercetin 3-(2G-xylosylrutinoside) were not detected in untreated KY140 but were present in treated KY140. Similarly, cinnamic acid and dihydromyricetin was not detected in untreated Atlantic but appeared in treated Atlantic. Conversely, (+)-afzelechin, (+)-catechin, and cosmetin were present in untreated KY140 but not detected in treated KY140. The DEGs encoding PAL, CYP73A, 4CL, C3’H, CAD, COMT, F5H, and CYP75B1 were upregulated in both Atlantic_vs_CS-Atlantic and KY140_vs_CS-KY140. The gene *StPAL* (*Soltu.Atl.09_2G005110*) encodes enzymes responsible for cinnamic acid biosynthesis. The genes *StPAL(Soltu.Atl.09_2G005110), St4CL(Soltu.Atl.03_3G030960)* and *StCYP73A*(including *Soltu.Atl.06_4G021440*, *Soltu.Atl.06_4G021450*, and *Soltu.Atl.06_1G015640*) were upregulated in both Atlantic_vs_CS-Atlantic and KY140_vs_CS-KY140. Conversely, *StCSE*(*Soltu.Atl.04_4G005370*) was downregulated in both Atlantic_vs_CS-Atlantic and KY140_vs_CS-KY140. These findings suggest that these genes may be involved in the regulation of cold resistance in potato.

### 3.10 “Linoleic acid metabolism” and “α-linolenic acid metabolism” in potato under CS

**Figure 10:**
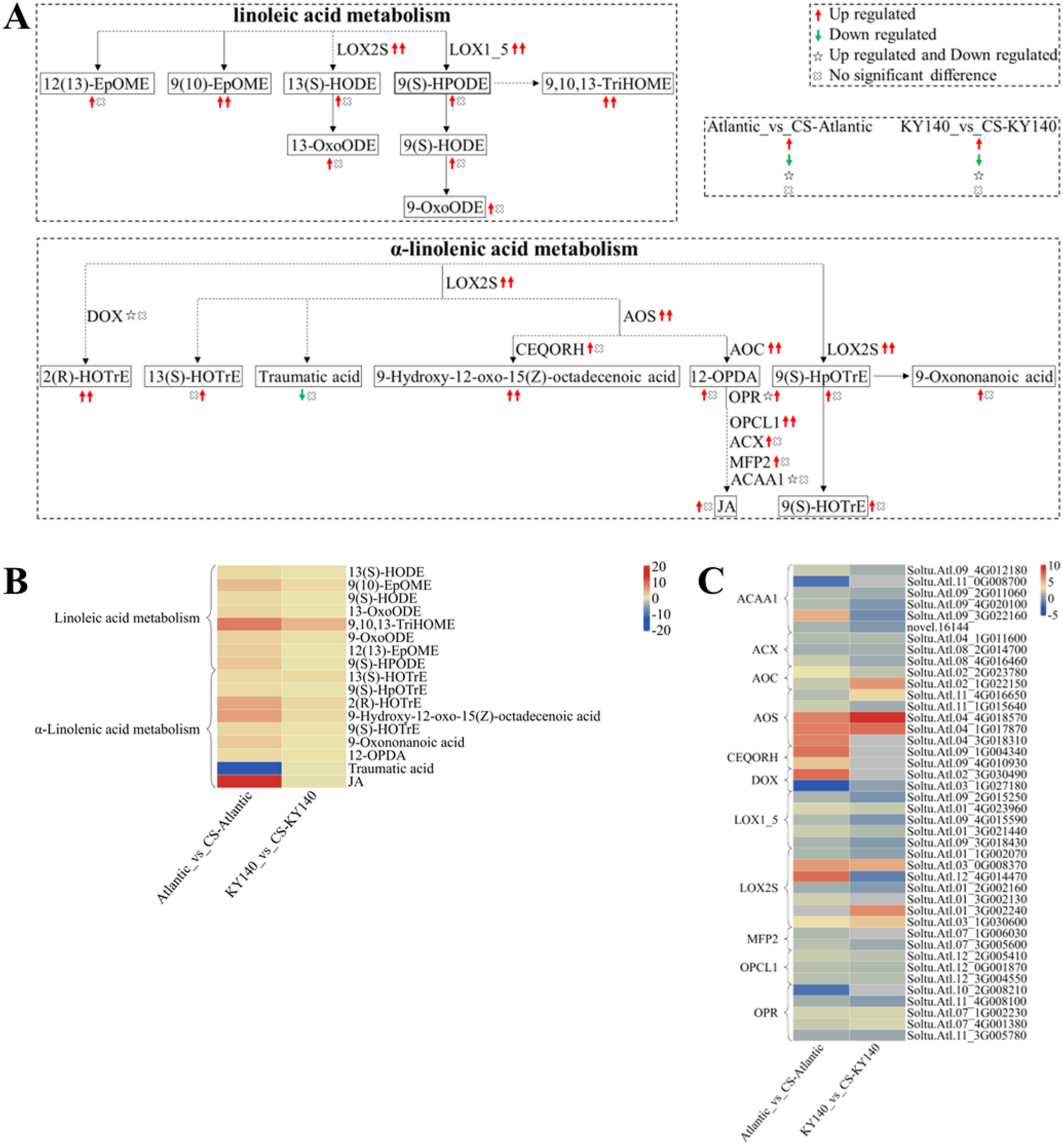
Metabolome and transcriptome profiling of differentially accumulated metabolites (DAMs) and differentially expressed genes (DEGs) associated with “linoleic acid metabolism” and “α-linolenic acid metabolism” in potato under cold stress (CS). **(A)** Comprehensive analysis of DAMs and related key enzymes in “linoleic acid metabolism” and “α-linolenic acid metabolism.” The ends of the arrow-headed lines represent DAMs, and the onlines represent enzymes. A solid line between two DAMs indicates a direct connection, while a dashed line indicates that there are other DAMs in between two DAMs. LOX2S, Lipoxygenase; LOX1_5, Linoleate 9S-lipoxygenase; DOX, Fatty acid α-dioxygenase; AOS, Hydroperoxide dehydratase; CEQORH, Chloroplastic oxoene reductase; AOC, Allene oxide cyclase; OPR, 12-Oxophytodienoic acid reductase; OPCL1, OPC-8:0 CoA ligase 1; ACX, Acyl-CoA oxidase; MFP2, Enoyl-CoA hydratase/3-hydroxyacyl-CoA dehydrogenase; ACAA1, Acetyl-CoA acyltransferase 1. DAMs and DEGs were investigated using the Kyoto Encyclopedia of Genes and Genomes (KEGG) to map the possible KEGG pathway maps for the biological interpretation of systemic functions (https://www.kegg.jp/kegg/pathway.html). **(B)** Heatmap of DAMs in “linoleic acid metabolism” and “α-linolenic acid metabolism.” The x-axis represents Atlantic_vs_CS-Atlantic_Log_2_FC and KY140_vs_CS-KY140_Log_2_FC. The colored bar in the upper right corner represents the contents of DAMs, with red indicating high-content DAMs and blue indicating low-content DAMs. **(C)** Heatmap of DEGs in “linoleic acid metabolism” and “α-linolenic acid metabolism.” The x-axis represents Atlantic_vs_CS-Atlantic_Log_2_FC and KY140_vs_CS-KY140_Log_2_FC. A color bar is shown in the upper right corner, with colors ranging from blue to red indicating the expression levels of DEGs from low to high.

To better analyze the synergy between DAMs and DEGs, we selected several DAMs and KEGG pathways related to “linoleic acid metabolism” and “α-Linolenic acid metabolism.” We constructed a schematic map indicating the differences between Atlantic and KY140 subjected to CS (**Figure 10**). We found elevated levels of 9(10)-EpOME, 9,10,13-TriHOME, 2(R)-HOTrE, and 9-hydroxy-12-oxo-15(Z)-octadecenoic acid in Atlantic_vs_CS-Atlantic and KY140_vs_CS-KY140, suggesting that they might participate in the regulation of potato resistance to CS. In addition, traumatic acid was detected in untreated Atlantic but not in treated Atlantic. In contrast, (-)-jasmonate(JA) was not detected in untreated Atlantic but appeared in treated Atlantic. The DEGs encoding LOX2S, LOX1_5, AOS, AOC, and OPCL1 were upregulated in both Atlantic_vs_CS-Atlantic and KY140_vs_CS-KY140. The genes *StAOC(Soltu.Atl.02_2G023780)*, *StLOX1_5(Soltu.Atl.01_4G023960)*, *StLOX2S(Soltu.Atl.03_0G008370)*, and *StOPCL1(Soltu.Atl.12_2G005410* and *Soltu.Atl.12_3G004550)*, were upregulated in both Atlantic_vs_CS-Atlantic and KY140_vs_CS-KY140, suggesting their involvement in the regulation of cold resistance in potato.

### 3.11 “Amino acid metabolism” in potato under CS

**Figure 11:**
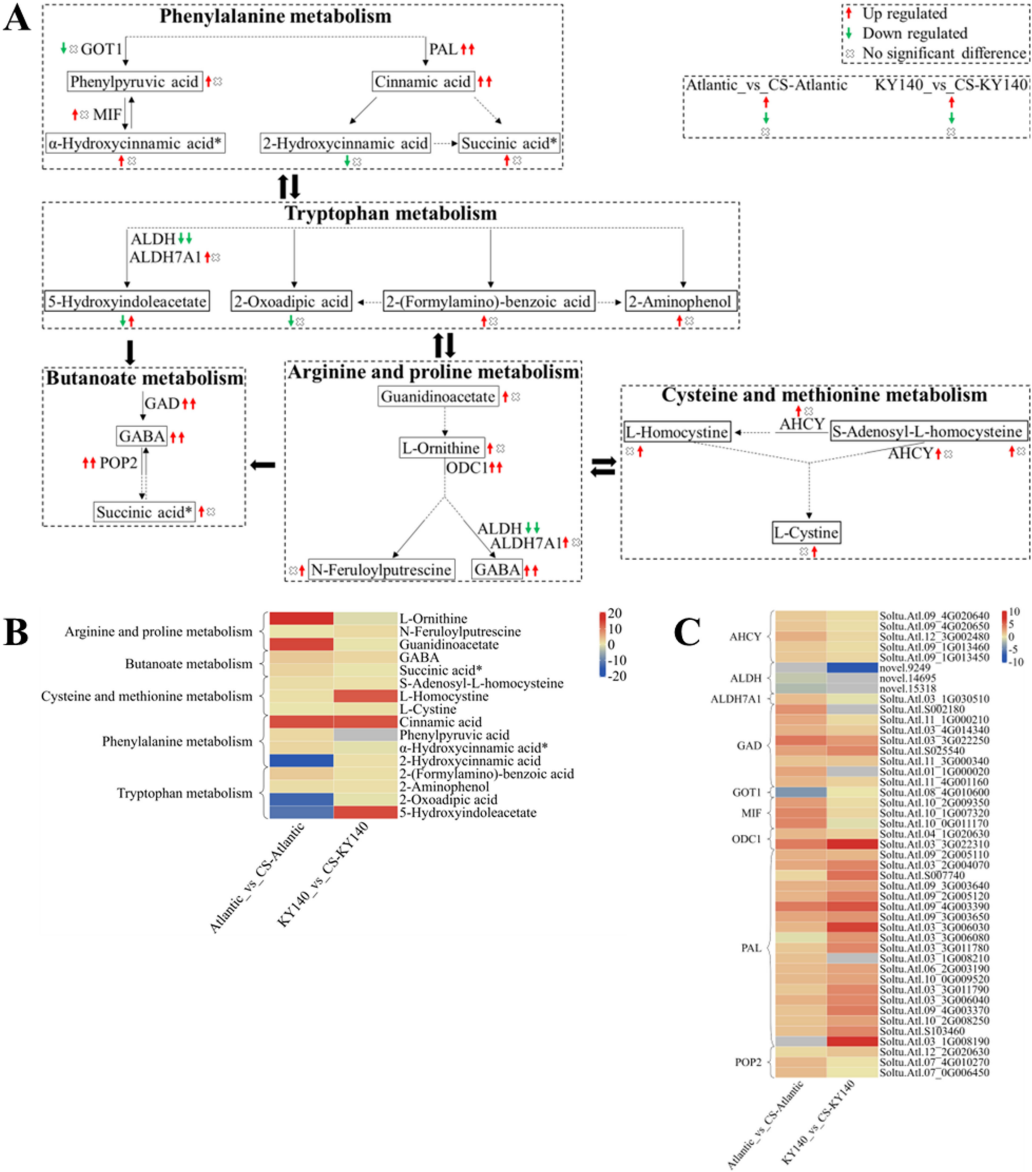
Metabolome and transcriptome profiling of differentially accumulated metabolites (DAMs) and differentially expressed genes (DEGs) associated with “amino acid metabolism” in potato under cold stress (CS). **(A)** Comprehensive analysis of DAMs and related key enzymes in “amino acid metabolism.” The ends of the arrow-headed lines represent DAMs, and the onlines represent enzymes. A solid line between two DAMs indicates a direct connection, while a dashed line indicates that there are other DAMs in between the two DAMs. GOT1, Aspartate aminotransferase, cytoplasmic; MIF, Phenylpyruvate tautomerase (double arrows indicate that MIF catalyzes a reversible reaction); PAL, Phenylalanine ammonia-lyase; ALDH, Aldehyde dehydrogenase (NAD^+^); ALDH7A1, Aldehyde dehydrogenase family 7 member A1; GAD, Glutamate decarboxylase; POP2, 4-Aminobutyrate-pyruvate transaminase (double arrows indicate that POP2 catalyzes a reversible reaction); ODC1, Ornithine decarboxylase 1; and AHCY, Adenosylhomocysteinase. DAMs and DEGs were investigated using the Kyoto Encyclopedia of Genes and Genomes (KEGG) to map the possible KEGG pathway maps for the biological interpretation of systemic functions (https://www.kegg.jp/kegg/pathway.html). **(B)** Heatmap of DAMs in “amino acid metabolism.” The x-axis represents Atlantic_vs_CS-Atlantic_Log_2_FC and KY140_vs_CS-KY140_Log_2_FC. When DAMs appear in multiple pathways, only one of the pathways is displayed, and the colored bar in the upper right corner represents the contents of DAMs, with red indicating high-content DAMs and blue indicating low-content DAMs. **(C)** Heatmap of DEGs in “amino acid metabolism.” The x-axis represents Atlantic_vs_CS-Atlantic_Log_2_FC and KY140_vs_CS-KY140_Log_2_FC. A color bar is shown in the upper right corner, with colors ranging from blue to red indicating the expression levels of DEGs from low to high.

To better analyze the synergy between DAMs and DEGs, we selected several DAMs and KEGG pathways related to “Amino acid metabolism” and constructed a schematic map indicating the differences between Atlantic and KY140 subjected to CS (**Figure 11**). 2-Hydroxycinnamic acid, 5-hydroxyindoleacetate, and 2-oxoadipic acid were present in untreated Atlantic but were not detected in treated Atlantic. 5-Hydroxyindoleacetate and L-Homocystine was not detected in untreated KY140 but appeared in treated KY140. γ-aminobutyric acid (GABA) levels were elevated in both Atlantic_vs_CS-Atlantic and KY140_vs_CS-KY140, suggesting that it might participate in the regulation of potato resistance to CS. Cinnamic acid was not detected in the untreated groups but was present in the treated groups, suggesting that it might also participate in the regulation of potato resistance to CS. Neither guanidinoacetate nor L-ornithine were detected in untreated Atlantic, but both were present in treated Atlantic. The DEGs encoding PAL, GAD, POP2, and ODC1 were upregulated in both treated groups. The gene *StGAD(Soltu.Atl.11_3G000340)*, which encodes enzymes involved in GABA biosynthesis, exhibited upregulation in both Atlantic_vs_CS-Atlantic and KY140_vs_CS-KY140. This suggests its potential role in regulating cold resistance in potato.

## 4 Discussion

### 4.1 Antioxidant enzymes and osmotic substances confer better cold resistance in potato

Low temperature significantly impacts potato. Our results showed that CS led to changes in antioxidant enzymes and osmotic substances. After CS treatment, Atlantic and KY140 exhibited varying physiological indicators responding to CS. For instance, Atlantic exhibited higher POD, CAT, and SOD activities and proline and soluble protein and sugar contents than KY140. CAT activity and soluble protein and sugar contents peaked at 12h in treated Atlantic, with the values higher than those in the untreated Atlantic. Moreover, SOD activity and proline content reached their lowest values at 12h in treated KY140, with lower values in treated KY140 than those in untreated KY140. Based on the comprehensive analysis of the field low-temperature period (usually 12h) and physiological indicators, the sampling time points of the potato metabolome and transcriptome and the qRT-PCR time points of DEGs were both 12 h after CS exposure. High activities of POD, CAT, and SOD with high contents of proline and soluble protein and sugar could enhance the cold resistance of potato (Xu et al., 2016; Wang M. X. et al., 2021; Duan et al., 2022).

### 4.2 Extensive targeted metabolomic analysis shows that several metabolites are involved in the regulation of cold resistance in potato

Low temperature can not only alter permeable substances but also cause a series of changes in metabolites. A total of 298 and 195 DAMs were identified in Atlantic and KY140, respectively. Among them, 124 DAMs were common between Atlantic and KY140, including lipids, flavonoids, alkaloids, organic acids, amino acids and their derivatives, nucleotides and their derivatives, lignans and coumarins, phenolic acids, and terpenoids. Overall, these DAMs showed an upward trend in both Atlantic_vs_CS-Atlantic and KY140_vs_CS-KY140. Zhang (2023) conducted an extensive untargeted metabolite analysis using eight potato materials based on liquid chromatography-mass spectrometry (LC-MS) for samples subjected to cold acclimation for one and 7 days. The detected metabolites were mainly associated with lipids, carboxylic acids, carbohydrates, amino acids, terpenoids, coumarins, and flavonoids.

One hundred DAMs (including 67 lipid-based DAMs) were found to participate in the regulation of potato resistance to CS. Previously, lipid accumulation was observed in cold-stressed maize (*Zea mays* L.), rice (*Oryza sativa* L.), and rapeseed (*Brassica napus* L.) (Zhao et al., 2021; Liu et al., 2022; Raza et al., 2021).

A total of 13 unsaturated fatty acids (UFAs) were differentially accumulated in both Atlantic_vs_CS-Atlantic and KY140_vs_CS-KY140. Among them, 11 UFAs might participate in the regulation of potato resistance to CS. In a previous study, multivariate statistical analyses revealed that the contents of UFAs in ‘Sanguinello’ (cold-resistant) were significantly higher than those in ‘Moro’ (cold-sensitive) (Habibi et al., 2021). Thus, the cold resistance of potato is mediated by the combined actions of various substances, not just one type of substance.

### 4.3 CS can cause dramatic changes in potato at the transcriptional level

A total of 8225 DEGs were detected in Atlantic_VS_CS-Atlantic and KY140_VS_CS-KY140, including 1131 common DEGs. Among them, 4751 and 1945 DEGs were upregulated and 2177 and 483 DEGs were downregulated in Atlantic_VS_CS-Atlantic and KY140_VS_CS-KY140, respectively. The differences between the two materials at the transcriptional level are primarily attributed to CS and the genetic background of the materials themselves. Among the 8225 DEGs, only 6869 DEGs were annotated to GO terms and categorized into 42 GO terms, including “biological process,” “molecular function,” and “cellular component.” In addition, we detected several differentially expressed TFs in Atlantic_vs_CS-Atlantic and KY140_vs_CS-KY140. These TFs belonged to the AP2/ERF, WRKY, MYB, bHLH, HB, bZIP, and NAC families (n = 85, 64, 64, 55, 44, 42, and 38, respectively). Among these TFs, 469 and 258 TFs were upregulated and 161 and 28 TFs were downregulated in Atlantic_vs_CS-Atlantic and KY140_vs_CS-KY140, respectively. In the Atlantic_vs_CS-Atlantic and KY140_vs_CS-KY140, the number of upregulated TFs significantly exceeded the number of downregulated TFs. The unique TFs might participate in the regulation of potato resistance (479 vs 135).

Similar results were obtained in wheat, where the TFs belonging to MADS, WRKY, ERF, bHLH, C_2_H_2_, MYB, and B3 families were involved in responses to CS (Lv et al., 2022). The most responsive TFs in Atlantic_vs_CS-Atlantic and KY140_vs_CS-KY140 belonged to the AP2/ERF family. This family was also involved in CS tolerance in *Poncirus trifoliata*. The α, β-amylase-encoding gene *PtrBAM1* of *Poncirus trifoliata* is regulated by CBF and plays an important role in cold resistance by regulating soluble sugar levels (Peng et al., 2014). The second most responsive TF families in Atlantic_vs_CS-Atlantic and KY140_vs_CS-KY140 were WRKY and MYB, which are also involved in the CS responses of other plants. The overexpression of KoWRKY40 (from *Kandelia obovata*) in *Arabidopsis* led to elevation in proline content and SOD, POD, and CAT activities (Fei et al., 2022). Under CS, *BpMYB4* overexpression in *Betula platyphylla* Suk. has been shown to increase SOD, POD, and CAT activities and soluble sugar and protein and proline contents (Zhang et al., 2019). The third most responsive TF family in Atlantic_vs_CS-Atlantic and KY140_vs_CS-KY140 was bHLH, which has been shown to be involved in the CS responses of other plants. Many *CsbHLH* genes in sweet orange (*Citrus sinensis*) respond to low temperatures, and knocking down *bHLH18* in trifoliate orange promotes cold sensitivity, accompanied by a downregulation of antioxidant genes and accumulation of more reactive oxygen. In contrast, *CsbHLH18* overexpression in transgenic tobacco enhanced its cold tolerance, with a significant reduction in the accumulation of reactive oxygen and an increase in the activity and transcriptional levels of antioxidant enzymes (Geng and Liu, 2018). Moreover, other TF families have also been shown to be involved in the regulation of plant cold resistance (Liu et al., 2014).

### 4.4 “Flavonoid-related metabolism” plays key roles in cold stress in potato 

We detected an elevation in the contents of cinnamic acid, caffeic acid, and naringenin in both Atlantic_vs_CS-Atlantic and KY140_vs_CS-KY140, suggesting their role in the regulation of potato resistance to CS. In Atlantic_vs_CS-Atlantic, prunin content decreased, but (+)-afzelechin contents were comparable. In KY140_vs_CS-KY140, prunin levels remained comparable while (+)-afzelechin levels decreased. Flavonoids, a product of phenylpropanoid metabolism, are essential substances in plants that influence their basic metabolism and stress resistance. Flavonoids are categorized into flavones, flavonols, flavanones, isoflavones, etc (Dong and Lin, 2021). Flavonoids has antioxidant and free radical scavenging properties (Zhang and Lu, 2006). The accumulation of flavonoids can enable plants to adapt to low-temperature environments (Sun et al., 2021; Yang et al., 2023). The upregulation of some flavonoid-related metabolites has been shown to play important roles in the CS responses of kiwifruit and peach (Sun et al., 2021; Li et al., 2023). Upon exposure to CS, wheat exhibits an increase in cinnamic acid and caffeic acid levels and a decrease in naringenin, (+)-afzelechin, and prunin levels (Lv et al., 2022). Caffeic acid has antioxidant and free radical scavenging properties due to the ortho-diphenol hydroxyl group on its own benzene ring (Bao et al., 2018). Any potential discrepancies between our results and the findings of previous studies might be attributed to conservation and selection during evolution.

In Atlantic_vs_CS-Atlantic and KY140_vs_CS-KY140, the DEGs encoding PAL, CYP73A, C3’H, F5H, COMT, CAD, 4CL, and CYP75B1 were upregulated. In Atlantic_vs_CS-Atlantic, the DEGs associated with CSE and CYP81E were both upregulated and downregulated. In KY140_vs_CS-KY140, the DEGs associated with CSE were downregulated, while those associated with CYP81E were upregulated. In Atlantic_vs_CS-Atlantic, the FLS-expressed DEGs were upregulated, while in KY140_vs_CS-KY140, these DEGs were both upregulated and downregulated. In Atlantic_vs_CS-Atlantic, the DEGs encoding CHI, CHS, F3H, DFR, and CYP75A were downregulated, while the expression levels of DEGs encoding FG2 were both upregulated and downregulated. These six enzymes were uniquely altered in Atlantic_vs_CS-Atlantic. Among various plant species, PAL is encoded by members of a multigene family that exhibit diverse expression patterns and play different roles in phenylpropanoid metabolism (Liu et al., 2018). Upregulation of PALs and COMT in cold-resistant peanuts might enhance cold resistance (Wang X. et al., 2021). Under CS, 59 out of 83 genes in the phenylpropanoid/flavonoid metabolic pathways (including Ko00940, Ko00941, and Ko00944) were significantly upregulated in wheat, including those encoding CHI, CHS, CYP73, 4CL, PAL, and CSE. Additionally, CYP75B1 plays a crucial role in two pathways (Ko00941 and Ko00944) associated with CS responses in wheat (Lv et al., 2022). Transcriptome analysis of the peach cultivars ‘Donghe No.1’ (cold-tolerant) and ‘21^st^ Century’ (cold-sensitive) under low-temperature treatment revealed upregulation of some key genes related to the biosynthetic of flavonoids in peach. These genes might play an important role in the responses of peach to CS, including those encoding COMT, CCR, CAD, PER, and F3’H. Their levels were significantly higher in ‘Donghe No.1’ than in ‘21^st^ Century’ under low-temperature stress (Li et al., 2023). Any potential discrepancies between our results and the findings of previous studies might be attributed to conservation and selection during evolution.

### 4.5 “Linoleic acid metabolism” and “α-linolenic acid metabolism” response to cold stress in potato 

In this study, “linoleic acid metabolism” and “α-linolenic acid metabolism” were enriched in both materials, regardless of whether they were analyzed by metabolomics or transcriptomics. With respect to these two pathways, more DAMs and DEGs were detected in Atlantic_vs_CS-Atlantic. The DEGs encoding LOX2S, LOX1_5, and AOS were upregulated in both Atlantic_vs_CS-Atlantic and KY140_vs_CS-KY140. The DEGs encoding OPR were both upregulated and downregulated in Atlantic_vs_CS-Atlantic and upregulated in KY140_vs_CS-KY140. The DEGs encoding ACX was upregulated in only Atlantic_vs_CS-Atlantic. Thus, ACX was uniquely altered in Atlantic. Although JA levels increased in both materials after CS, the increase in KY140_vs_CS-KY140 was not significant. JA was not detected in untreated Atlantic but appeared in treated Atlantic.

Stable lipid metabolism can not only reduce ROS accumulation but also relieve oxidative stress in the cells (Patel et al., 2016; Xu et al., 2023). Xu et al. (2023) conducted omics analysis on ZQ (cold-sensitive) and XL (cold-tolerant) qingke varieties under low temperatures. Both transcriptomic and metabolomic analyses showed that linoleic acid metabolism and other lipid metabolisms were enriched in XL. The transcriptome analysis of naturally cold-acclimated ‘Elsie Lee’ by Liu B. et al. (2020) also enriched “linoleic acid metabolism” and “α-linolenic acid metabolism.” Korean scholars analyzed ACXs expression patterns in rice subjected to mechanical injury and speculated that OsACX2 provided sugar and energy for germinating seeds, while OsACX1 was involved in mechanical injury response and JA synthesis (Kim et al., 2007). Cold temperatures can induce JA accumulation in rice (Du et al., 2013). JA is an upstream signal of the ICE-CBF/DREB1 pathway, and its exogenous application can positively regulate freezing tolerance in *Arabidopsis* (Hu et al., 2013). JA participates in the regulation of phenylpropanoid metabolism, such as flavonoid synthesis (Dong and Lin, 2021). α-linolenic acid and linoleic acid are polyunsaturated fatty acids (PUFAs) and are substrates of LOX (Porta and Rocha-Sosa, 2002). PUFAs can maintain membrane fluidity (Sakai and Larcher, 1987) and accumulate during CS (Vega et al., 2004; Upchurch, 2008). AtLOX2 and AtCYP74A(AOS) are key enzymes impacting JA biosynthesis in *Arabidopsis* (Bell et al., 1995; Spoel et al., 2003; Park et al., 2002). The DEGs encoding LOX are involved in both “linoleic acid metabolism” and “α-linolenic acid metabolism” and are downregulated in field cold acclimation (CA). 13-LOXs and AOSs were downregulated in both field CA and artificial CA. DEGs annotated as 12-OPR3 showed very low transcription levels or were downregulated in field CA. JA content decreased in field CA and artificial CA. Inhibiting JA biosynthesis and suppressing the consumption of linolenic acid might help maintain the membrane fluidity under CS (Liu B. et al., 2020). We proposed promoting JA accumulation to make potato resistant to cold, and the cold resistance mechanism of ‘Elsie Lee’ was different from that speculated in the current study. Any potential discrepancies between our results and the findings of previous studies might be attributed to conservation and selection during evolution. The “Linoleic acid metabolism” and “α-Linolenic acid metabolism” were found to be more active in Atlantic, potentially mediating their enhanced cold resistance. The specific roles of JA in regulating CS responses in potato remain to be fully elucidated and can be further explored through exogenous application of JA and genetic engineering approaches.

### 4.6 The crucial role of “amino acid metabolism” in stress tolerance

We found GABA elevation in Atlantic_vs_CS-Atlantic and KY140_vs_CS-KY140, suggesting its role in the regulation of potato resistance. Succinic acid* was upregulated in Atlantic_vs_CS-Atlantic, however, it was not significantly different in KY140_vs_CS-KY140. Furthermore, the expression of GAD DEGs increased in both Atlantic_vs_CS-Atlantic and KY140_vs_CS-KY140. GABA, a four-carbon non-protein amino acid, can regulate the pH of the intracellular and extracellular environment, maintain the balance of carbon and nitrogen, and participate in the response of plants to abiotic stresses, and is considered a plant molecular signal (Ramesh et al., 2017). The combination of low-temperature acclimation and ice-cold storage can activate the GABA shunt metabolism in the flesh of longan fruits, leading to GABA accumulation (Zhou, 2017). Exogenous GABA treatment and GABA combined with GABA-T inhibitor (VGB) treatment effectively inhibited the occurrence of cold injury in peach fruits during low-temperature storage by increasing the endogenous GABA and sucrose contents in the later stages of peach (*Prumus persica* L.) fruit storage (Fang et al., 2020). Exogenous GABA treatment of cucumber seedlings under CS can improve their antioxidant enzyme activity, reduce the damage of CS to cucumber seedlings, and facilitate the normal growth of cucumber seedlings (Huang, 2015). Exogenous GABA treatment of peach fruit increased endogenous GABA and enhanced *PpGAD* expression, enhanced cold resistance of the fruit, and prevented the occurrence of cold damage in peach fruit (Song et al., 2016). Overexpression of rice (*Oryza sativa* L.) *GAD2* gene can significantly increase the endogenous GABA content in rice (Shimajiri et al., 2013). Under CS conditions, succinic acid* was downregulated in the phenylalanine metabolism of two rapeseed varieties (Raza et al., 2021). Any potential discrepancies between our results and the findings of previous studies might be attributed to conservation and selection during evolution. Overall, the “amino acid metabolism” was more active in Atlantic, which might be related to its enhanced cold resistance. Whether GABA can enhance cold tolerance in potato can be further validated through exogenous application and genetic regulation (overexpression or knockout of related genes).

## 5 Conclusion

The differences between Atlantic (cold-resistant) and KY140 (cold-sensitive) potato varieties were analyzed from the aspects of physiology, metabolomics, and transcriptomics. Atlantic exhibited higher POD, CAT, and SOD activities and proline and soluble protein and sugar contents than KY140 under different treatments. More DAMs and DEGs are induced in Atlantic after CS. The intrinsic mechanisms between the biosynthesis of metabolites and gene expression were discussed. We found that the metabolites of lipids, flavonoids, alkaloids, organic acids, amino acids and their derivatives, nucleotides and their derivatives, lignans and coumarins, phenolic acids, and terpenoids, as well as the metabolic pathways of “flavonoid-related metabolism,” “lipid metabolism,” “amino acid metabolism,” “carbohydrate metabolism,” “nucleotide metabolism,” and “energy metabolism” might play an important role in the cold resistance of potato. The key regulatory DAMs, DEGs, and their roles in cold resistance via the “flavonoid-related metabolism,” “lipid metabolism,” and “amino acid metabolism” need to be further explored and validated.

## Data Availability

The datasets utilized in this research are publicly available in online repositories. The repository/repositories and corresponding accession number(s) can be accessed at the following: https://www.ncbi.nlm.nih.gov/geo/query/acc.cgi?acc=GSE291340.
