# Correction: Metabolome and transcriptome analyses reveal molecular responses of two potato (*Solanum tuberosum* L.) cultivars to cold stress

**DOI:** 10.3389/fpls.2025.1635281

**Published:** 2025-07-03

**Authors:** Xiang Li, Zhenzhen Zheng, Yun Zhou, Shenglong Yang, Wang Su, Heng Guo, Guangji Ye, Jian Wang

**Affiliations:** ^1^ Academy of Agriculture and Forestry Sciences, Qinghai University, Xining, China; ^2^ Qinghai Provincial Key Laboratory of Potato Breeding, Qinghai University, Xining, China; ^3^ Ministry of Education Engineering Research Center of Potato in Northwest Region, Qinghai University, Xining, China

**Keywords:** potato (*Solanum tuberosum L.*), cold stress (CS), differentially accumulated metabolites(DAMs), differentially expressed genes (DEGs), KEGG pathway

In the published article, there was an error in **Figure 9A**, **Figure 10A** and **Figure 11A** as published. The labels in the upper right corners of **Figure 9A**, **Figure 10A** and **Figure 11A** contain inaccuracies. “No significant difference or no detection” has been corrected to “No significant difference” “CK/0h” has been corrected to “Atlantic_vs_CS-Atlantic” “CS/12h” has been corrected to “KY140_vs_CS-KY140”. The corrected **Figures 9A**–**11A** and their captions appear below.

**Figure 9:**
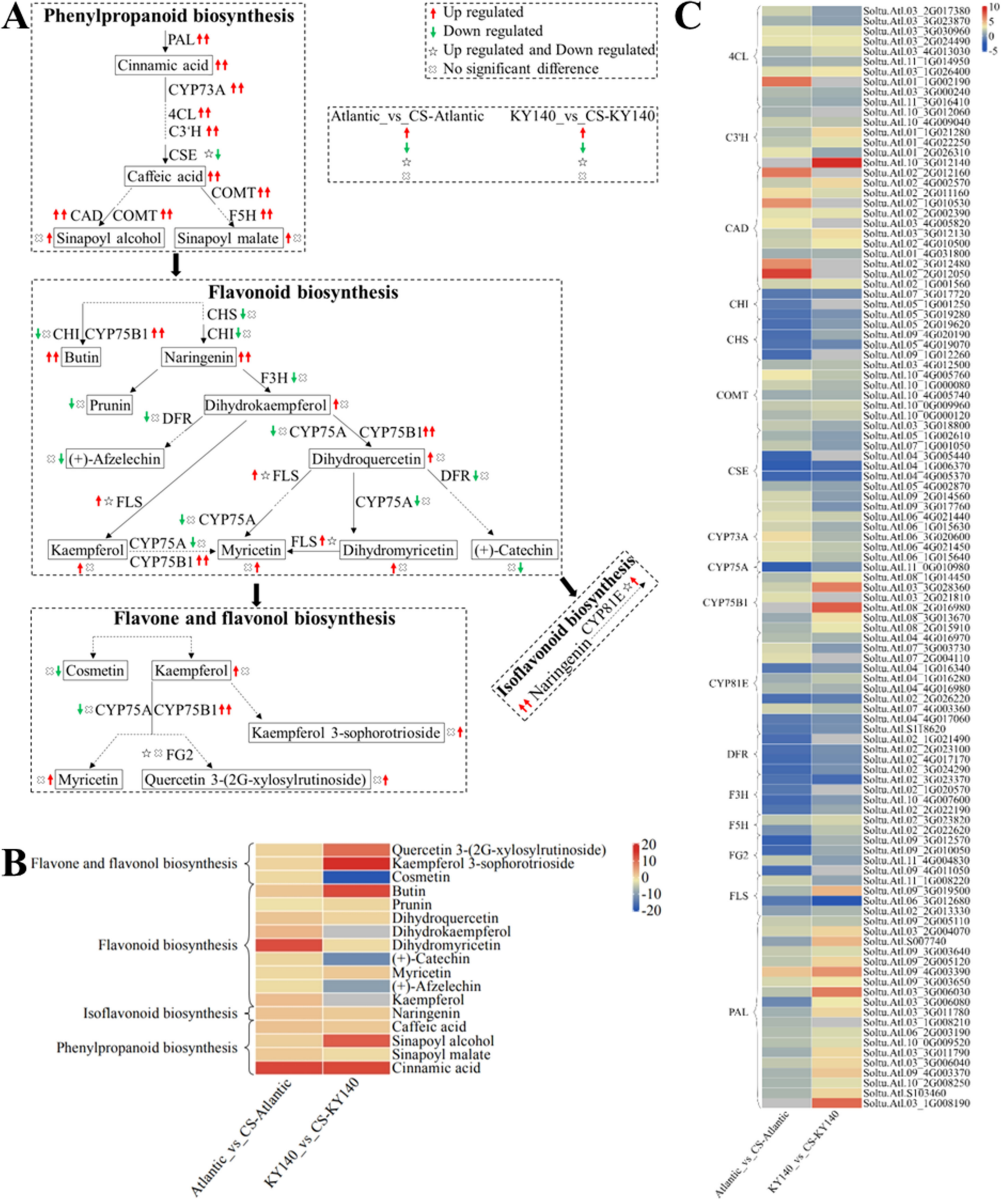
Metabolome and transcriptome profiling of differentially accumulated metabolites (DAMs) and differentially expressed genes (DEGs) associated with “flavonoid-related metabolism” in potato under cold stress (CS). **(A)** Comprehensive analysis of DAMs and related key enzymes in “flavonoid-related metabolism.” The ends of the arrow-headed lines represent DAMs, and the onlines represent enzymes. A solid line between two DAMs indicates a direct connection, while a dashed line indicates that the two DAMs are not directly connected but have other DAMs in between. PAL, Phenylalanine ammonia-lyase; C3’H, 5-O-(4-coumaroyl)-D-quinate 3’-monooxygenase; F5H, Ferulate-5-hydroxylase; CYP73A, Trans-cinnamate 4-monooxygenase; CSE, Caffeoyl shikimate esterase; COMT, Caffeic acid 3-O-methyltransferase; 4CL, 4-Coumarate: CoA ligase; CAD, Cinnamyl-alcohol dehydrogenase; F3H, Flavanone-3-hydroxylase; DFR, Bifunctional dihydroflavonol 4-reductase/flavanone 4-reductase; FLS, Flavonol synthase; CYP75B1, Flavonoid 3’-monooxygenase; CHI, Chalcone isomerase; CHS, Chalcone synthase; CYP75A, Flavonoid 3’,5’-hydroxylase; CYP81E, Isoflavone/4’-methoxyisoflavone 2’-hydroxylase; and FG2, Flavonol-3-O-glucoside L-rhamnosyltransferase. DAMs and DEGs were investigated using the Kyoto Encyclopedia of Genes and Genomes (KEGG) to map the possible KEGG pathway maps for the biological interpretation of systemic functions (https://www.kegg.jp/kegg/pathway.html). **(B)** Heatmap of DAMs in “flavonoid-related metabolism.” The x-axis represents Atlantic_vs_CS-Atlantic_Log_2_FC and KY140_vs_CS-KY140_Log_2_FC. When DAMs appear in multiple pathways, only one of the pathways is displayed, and the colored bar in the upper right corner represents the contents of DAMs, with red color indicating high-content DAMs and blue color indicating low-content DAMs. **(C)** Heatmap of DEGs in “flavonoid-related metabolism.” The x-axis represents Atlantic_vs_CS-Atlantic_Log_2_FC and KY140_vs_CS-KY140_Log_2_FC. A color bar is shown in the upper right corner, with colors ranging from blue to red indicating the expression levels of DEGs from low to high.

**Figure 10:**
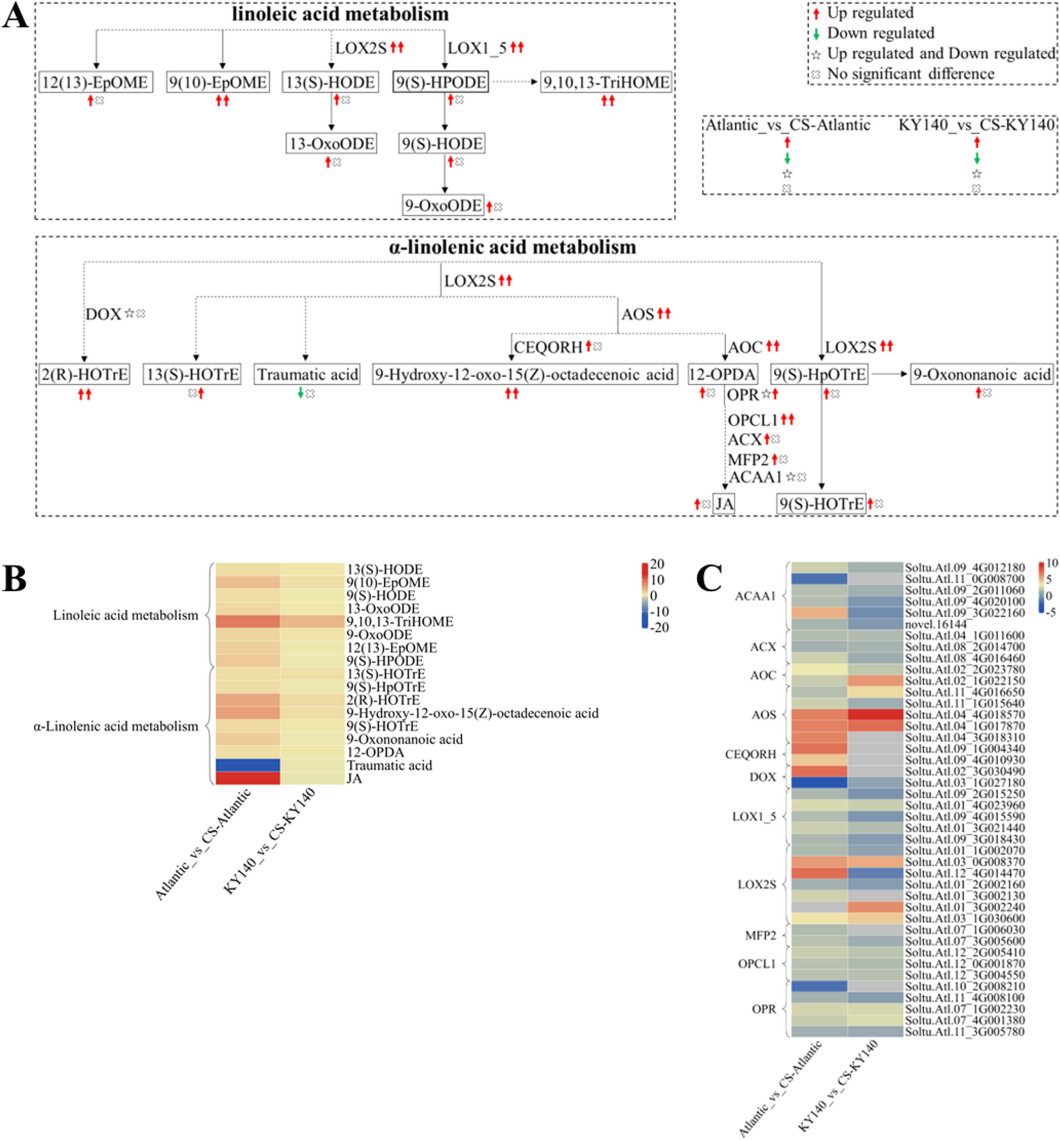
Metabolome and transcriptome profiling of differentially accumulated metabolites (DAMs) and differentially expressed genes (DEGs) associated with “linoleic acid metabolism” and “α-linolenic acid metabolism” in potato under cold stress (CS). **(A)** Comprehensive analysis of DAMs and related key enzymes in “linoleic acid metabolism” and “α-linolenic acid metabolism.” The ends of the arrow-headed lines represent DAMs, and the onlines represent enzymes. A solid line between two DAMs indicates a direct connection, while a dashed line indicates that there are other DAMs in between two DAMs. LOX2S, Lipoxygenase; LOX1_5, Linoleate 9S-lipoxygenase; DOX, Fatty acid α-dioxygenase; AOS, Hydroperoxide dehydratase; CEQORH, Chloroplastic oxoene reductase; AOC, Allene oxide cyclase; OPR, 12-Oxophytodienoic acid reductase; OPCL1, OPC-8:0 CoA ligase 1; ACX, Acyl-CoA oxidase; MFP2, Enoyl-CoA hydratase/3-hydroxyacyl-CoA dehydrogenase; ACAA1, Acetyl-CoA acyltransferase 1. DAMs and DEGs were investigated using the Kyoto Encyclopedia of Genes and Genomes (KEGG) to map the possible KEGG pathway maps for the biological interpretation of systemic functions (https://www.kegg.jp/kegg/pathway.html). **(B)** Heatmap of DAMs in “linoleic acid metabolism” and “α-linolenic acid metabolism.” The x-axis represents Atlantic_vs_CS-Atlantic_Log_2_FC and KY140_vs_CS-KY140_Log_2_FC. The colored bar in the upper right corner represents the contents of DAMs, with red indicating high-content DAMs and blue indicating low-content DAMs. **(C)** Heatmap of DEGs in “linoleic acid metabolism” and “α-linolenic acid metabolism.” The x-axis represents Atlantic_vs_CS-Atlantic_Log_2_FC and KY140_vs_CS-KY140_Log_2_FC. A color bar is shown in the upper right corner, with colors ranging from blue to red indicating the expression levels of DEGs from low to high.

**Figure 11:**
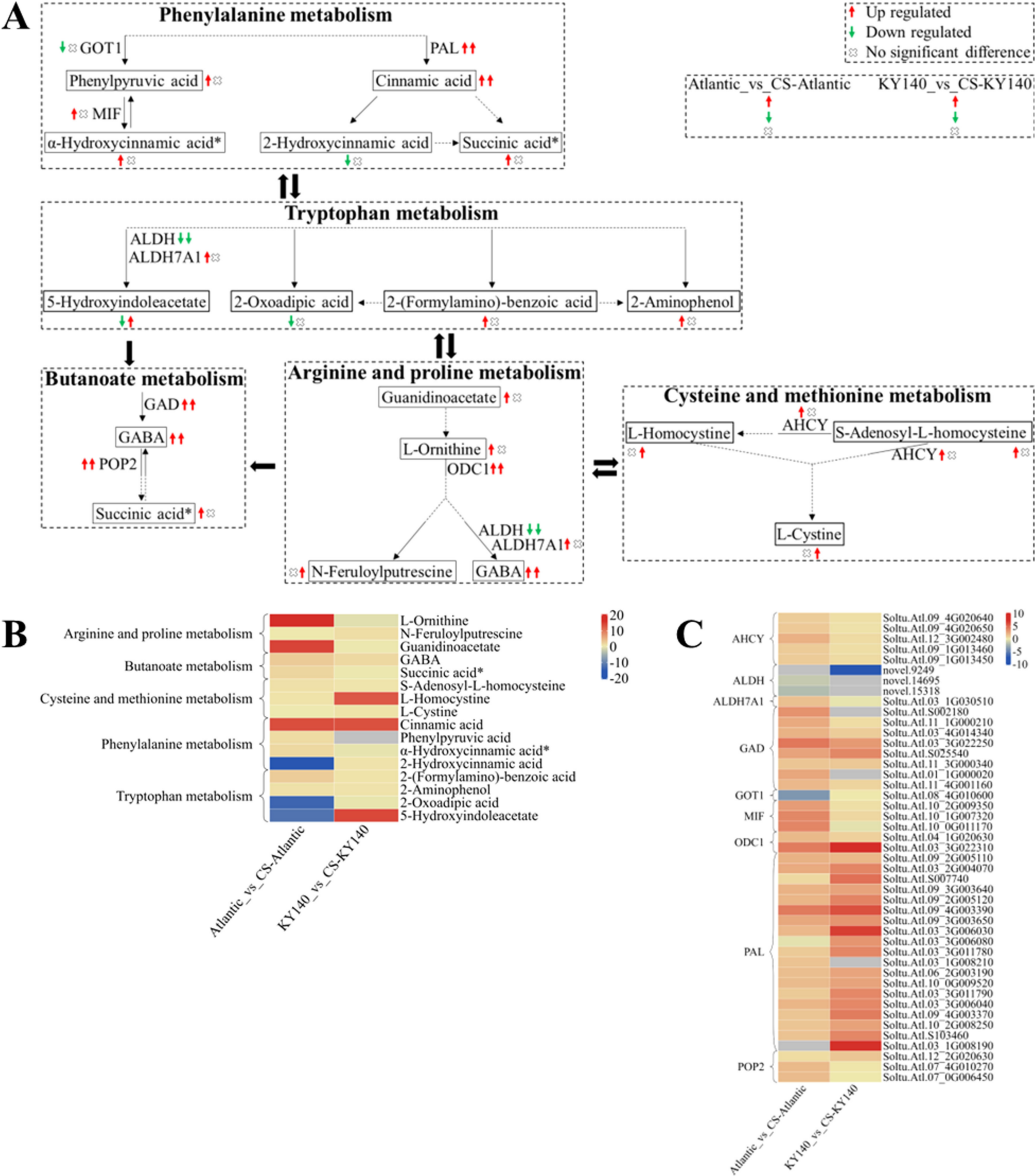
Metabolome and transcriptome profiling of differentially accumulated metabolites (DAMs) and differentially expressed genes (DEGs) associated with “amino acid metabolism” in potato under cold stress (CS). **(A)** Comprehensive analysis of DAMs and related key enzymes in “amino acid metabolism.” The ends of the arrow-headed lines represent DAMs, and the onlines represent enzymes. A solid line between two DAMs indicates a direct connection, while a dashed line indicates that there are other DAMs in between the two DAMs. GOT1, Aspartate aminotransferase, cytoplasmic; MIF, Phenylpyruvate tautomerase (double arrows indicate that MIF catalyzes a reversible reaction); PAL, Phenylalanine ammonia-lyase; ALDH, Aldehyde dehydrogenase (NAD^+^); ALDH7A1, Aldehyde dehydrogenase family 7 member A1; GAD, Glutamate decarboxylase; POP2, 4-Aminobutyrate-pyruvate transaminase (double arrows indicate that POP2 catalyzes a reversible reaction); ODC1, Ornithine decarboxylase 1; and AHCY, Adenosylhomocysteinase. DAMs and DEGs were investigated using the Kyoto Encyclopedia of Genes and Genomes (KEGG) to map the possible KEGG pathway maps for the biological interpretation of systemic functions (https://www.kegg.jp/kegg/pathway.html). **(B)** Heatmap of DAMs in “amino acid metabolism.” The x-axis represents Atlantic_vs_CS-Atlantic_Log2FC and KY140_vs_CS-KY140_Log2FC. When DAMs appear in multiple pathways, only one of the pathways is displayed, and the colored bar in the upper right corner represents the contents of DAMs, with red indicating high-content DAMs and blue indicating low-content DAMs. **(C)** Heatmap of DEGs in “amino acid metabolism.” The x-axis represents Atlantic_vs_CS-Atlantic_Log2FC and KY140_vs_CS-KY140_Log2FC. A color bar is shown in the upper right corner, with colors ranging from blue to red indicating the expression levels of DEGs from low to high.

The original article has been updated.

